# Nutrient environment improves drug metabolic activity in human iPSC-derived hepatocytes and HepG2

**DOI:** 10.1007/s00204-025-04139-4

**Published:** 2025-08-12

**Authors:** Victoria Pozo Garcia, Tuğçe S. Çobanoğlu, Helen Sophie Hammer, Rita Carlota, Kasper Holm, Catherine Verfaillie, Oliver Poetz, Paul Jennings, Sofia Moco

**Affiliations:** 1 Department of Chemistry and Pharmaceutical Sciences, Amsterdam Institute of Molecular and Life Sciences (AIMMS), Vrije Universiteit (VU) Amsterdam, De Boelelaan 1108, 1081 HZ Amsterdam, the Netherlands; 2 Signatope GmbH, Reutlingen, Germany; 3 Department of Development and Regeneration, Stem Cell Institute, KU Leuven, Leuven, Belgium; 4 NMI Natural and Medical Sciences Institute at the University of Tuebingen, Tuebingen, Germany

**Keywords:** Induced pluripotent stem cells (iPSCs), Drug metabolism, Metabolomics, Liver, Cytochrome P450 (CYP), LC–MS

## Abstract

Induced pluripotent stem cells (iPSCs) have emerged as a transformative tool in regenerative medicine, in liver research. The perspective of a stable and functional source of hepatocytes has led to developing protocols for human iPSC-derived hepatocytes-like cells (HLCs). Yet, hepatic models remain one of most challenging systems to functionally reproduce with iPSCs, due to its resulting limited metabolic function. Using an adapted nutrient regimen, two human hepatocyte models were characterized: HLCs (derived from iPSCs) and metabolically active HepG2 (mHepG2, derived from the cell line HepG2), for their drug metabolism activity. In these cell systems, the transcriptome, proteome, and metabolome of 11 drug-relevant cytochrome P450 (CYP) isoenzymes were studied. A liquid chromatography–mass spectrometry (LC–MS)-based metabolomics approach, using model drugs as isoenzyme reporters, was applied, achieving a comprehensive overview of mHepG2 and HLCs drug metabolism. Drugs used in this study to characterize xenobiotic machinery were: bupropion (25 µM), phenacetin (30 µM), rosiglitazone (10 µM), diclofenac (75 µM), dextromethorphan (15 µM), chlorzoxazone (60 µM), midazolam (15 µM), benzydamine (15 µM), coumarin (250 µM) and 7-ethoxycoumarin (60 µM). Being HepG2 notorious for its limited metabolic capacity, our study raises mHepG2 as a highly performant cell model, with activity on 8 drug-metabolizing CYPs. Modulation by nutrient environment in improving metabolic function of in vitro models is here proven as a key determinant. Likewise, HLCs hold the widest CYP coverage at the transcript level and were able to cope with a wide variety of chemical insults, making them a promising model for personalized metabolic studies.

**Supplementary Information:**

The online version contains supplementary material available at 10.1007/s00204-025-04139-4.

## Introduction

Induced pluripotent stem cells (iPSCs) have emerged over the past years as a promising model for personalized cell therapies and disease modeling (Aboul-Soud et al. 2021). Pluripotency and self-renewal are key iPSC characteristics for their application in regenerative medicine. Through differentiation protocols, patient-specific cells from all three germ layers (ectoderm, endoderm, and mesoderm) can be generated, maintaining patient genetic background, and epigenetics (Choudhury et al. 2017; Aboul-Soud et al. 2021). The applicability of iPSCs was already challenged in studying clinical diseases such as liver steatosis (Kozyra et al. 2018), Wilson’s disease, familial hypercholesterolemia (Hannoun et al. 2016), and alcohol-associated liver cirrhosis (Mukhopadhyay et al. 2024). Hence, iPSCs derived from patients with liver diseases can be used for pathophysiological studies, evaluating cellular response to therapeutic agents and unraveling disease-molecular mechanisms, to find new drug targets (Hannoun et al. 2016). Despite iPSCs’ potential, differentiation protocols often lead to immature cells retaining embryonic features (Choudhury et al. 2017; Li et al. 2019; Boon et al. 2020). This is commonly the case for hepatocytes-like cells (HLCs) derived from iPSCs, for which differentiation protocols result in cells with less metabolic activity compared to liver tissue (Li et al. 2019; Boon et al. 2020; Gupta et al. 2021).

A major function of the liver is to deal with xenobiotics. The great majority of drugs undergo CYP-mediated biotransformation in the liver (Guengerich 2005). The superfamily of CYPs includes 57 isoenzymes in humans, in which 3 families (1, 2 and 3) have central roles in phase I drug metabolism (Guengerich 2005). CYPs are also well known for drug bioactivation (Kalgutkar and Soglia 2005; Spaggiari et al. 2014; Esteves et al. 2021). A particular CYP isoform, CYP3A4, metabolizes ∼ 50% of prescription drugs (Guengerich 2005; Boon et al. 2020). Another important group of detoxification enzymes is part of phase II metabolism, mediating conjugations with sulfate, acetate, glutathione and glucuronate, resulting in mostly hydrophilic products (Jancova et al. 2010). UDP-glucuronosyltransferases (UGTs) and sulfotransferases (SULTs) are among the most human-relevant phase II enzymes, often associated with improving drug clearance and elimination (Jancova et al. 2010).

Drug metabolism studies are pivotal in drug development programs as the early identification of reactive metabolites, through bioactivation, is vital for preventing drug-induced liver injury (DILI) (David and Hamilton 2010; Chen et al. 2011; Fernandez-Checa et al. 2021). These electrophilic intermediates produced by drug metabolism may induce diverse toxicity pathways, such as DNA damage, oxidative stress, endoplasmic reticulum stress (unfolded protein response/integrated stress response), and/or mitochondrial dysfunction, associated with DILI (Holt and Ju 2006; David and Hamilton 2010). DILI remains one of the main reasons for drug withdrawal during clinical trials or even from the market (David and Hamilton 2010; Chen et al. 2011; Fernandez-Checa et al. 2021). DILI remains poorly predicted during preclinical animal testing due to interspecies limitations, which include differences in drug metabolism (Gómez-Lechón et al. 2016; Dirven et al. 2021).

Early drug metabolism testing is primarily performed in vitro. The human in vitro models HepG2 and HepaRG are tumor-derived cell lines commonly used in hepatotoxicity and intrinsic clearance studies (Stanley and Wolf 2022). However, HepG2 expresses low levels of many phase I enzymes, including CYP3A4 (Arzumanian et al. 2021; Yang et al. 2023). With lower expression of certain CYP isoenzymes, HepaRG has CYP3A4 expression; thus, it is generally considered a better cell model compared to HepG2 (Andersson et al. 2012). However, primary human hepatocytes (PHHs) and human liver microsomes (HLMs) remain the gold standard in drug metabolism studies, given their superior metabolic activity, even if perishable and of short-term use (Vorrink et al. 2017; Boon et al. 2020; Sun et al. 2024).

With the rise of novel PSCs technologies and differentiation protocols holding promise in enhancing metabolism in cultured hepatocytes in vitro, Boon and colleagues report a differentiation protocol, applied to a human embryonic stem cell (hESC) line, creating hepatic progeny expressing CYP3A4 (Boon et al. 2020). This approach led to an unprecedented boost in generalized metabolic function, achieved by supplementing the culture media with an excess concentration of amino acids. Improved expression and function were not only observed in HLCs derived from PSCs, but also in HepG2 cultured analogously—here referred as metabolically active HepG2, mHepG2 (Boon et al. 2020). However, a comprehensive characterization of drug metabolism in these systems was then not conducted.

The current study performs an in-depth characterization of the drug metabolism machinery of these two promising hepatic cell systems, HLCs (here derived from induced PSCs) and mHepG2, for their phase I and II metabolic capacities. A combination of transcriptomics, proteomics, and metabolomics was used for characterizing 11 drug-relevant CYP isoenzymes. Liquid chromatography–mass spectrometry (LC–MS)-based metabolomics was here developed to follow the widest range of drug biotransformation in each hepatic model, and metabolic activities were additionally tested in human liver microsomes (HLMs) as a benchmark in drug metabolism studies.

## Materials and methods

### Materials

Materials were listed in the Supplementary Information Tables S1–12.

### Cell culture

All cell systems were cultured in an incubator at 37 °C under a humidified atmosphere with 5% CO_2_.

#### HepaRG cells

Human hepatoma HepaRG were obtained from Biopredic International (Saint Grégoire, France) as undifferentiated cells. Cells were cultured in William’s E medium, supplemented with 2 mM GlutaMAX™, 1% (v/v) penicillin/streptomycin, 9% (v/v) fetal bovine serum (FBS), 50 µM hydrocortisone 21-hemisuccinate and 5 µg/mL human insulin. Cells were passaged with trypsin and seeded with a density of 45.000 cells/cm^2^. Forty-eight hours after seeding, cells were differentiated as previously described (Capinha et al. 2023), by supplementing the cultured media with 1.7% (v/v) DMSO for a week. Cells were used between passages 20 and 30 and tested 1 day after differentiation.

#### HepG2 cells

Human hepatoma HepG2 was purchased from European Collection of Authenticated Cell Cultures (ECACC, UK Health Security Agency). Cells were cultured in DMEM media with low glucose (5.5 mM) and pyruvate (1 mM), 1% (v/v) penicillin/streptomycin and 9% (v/v) FBS. Cells were passaged with trypsin and seeded with a density of 45.000 cells/cm^2^. Cells were used 48 h after seeding and were kept between passages 12 and 20.

#### mHepG2 (metabolically active HepG2) cells

HepG2 cells were metabolically matured by adding a high concentration of amino acids in the culture media, following the protocol proposed by Boon et al. 2020. Cells were seeded with a density of 45.000 cells/cm^2^ and 5 days after seeding (with a 70–80% well confluency), the media was supplemented with amino acids (AAHepG2 media: 16 mL of non-essential amino acids, 8 mL of essential amino acids solution which contained 100× and 50×, respectively, minimum essential medium’s (MEM) amino acids concentration, except L-glutamine, and additional 20 g/L of glycine in 100 mL HepG2 culture media, see Table S8). HepG2 were matured in AAHepG2 media for 22 days, before being used.

#### iPSCs culture and differentiation to HLCs (hepatocyte-like cells)

Human induced pluripotent stem cells (iPSCs) used in this study (SBAD2, STBCi321-A, https://www.cellosaurus.org/CVCL_ZX54) were generated during the IMI-funded StemBANCC project using the CytoTune 2.0 (Thermo Fisher) Sendai viral reprogramming kit from normal dermal fibroblasts (Morrison et al. 2015). These cells have been successfully differentiated into several phenotypes including hepatocyte-, lung-, neuronal- and renal-like cells (Chandrasekaran et al. 2024). SBAD2-3x iPSC line was generated with a donor vector containing Hepatocyte Nuclear Factor 1 Alpha (HNF1A), Forkhead Box A3 (FOXA3) and Prospero Homeobox Protein (PROX1), through a doxycycline inducible cassette as described elsewhere (Ghosh et al. 2021). The SBAD2-3x cell line authentication was conducted by Eurofins, matching the SBAD2 parent cell line. SBAD2-3x cells were cultured in chemically defined media, here referred as VU8 media (Naderlinger et al. 2025), see Table S9. iPSCs were cultured on the human embryonic stem cell-qualified reduced growth factor basement membrane matrix Geltrex™ (Geltrex) coating. Cultures were passaged twice per week, with ratio 1:6–1:8. iPSCs passaging, thawing and freezing protocols were followed as detailed elsewhere (Meijer et al. 2023).

iPSCs were differentiated to HLCs as previously described (Boon et al. 2020) with a few modifications. Shortly, SBAD2-3x were passaged with accutase and seeded at a density of 35.000 cells/cm^2^ in VU8 media with 10 µM Y-27632 dihydrochloride ROCK inhibitor on Geltrex-coated plates. Twenty-four hours after seeding, media was changed into LDM media, as previously described (Vanhove et al. 2016; Boon et al. 2020) with 10 µM of hydrocortisone 21-hemisuccinate, see Table S10. During the first 12 days of differentiation, media was supplemented with the following cytokines (see Fig. S1, Table S11): 0.05 µg/mL Wnt3a (day 0), 0.05 µg/mL Activin A (day 2), 0.05 µg/mL BMP4 (day 4–8) and 0.02 µg/mL FGF1 (day 8–12) with 0.6% (v/v) DMSO. At day 12, high concentration of amino acids was added to the media (AA3 media, see Table S8) with 0.02 µg/mL HGF and 2% (v/v) DMSO. From day 14, glycine (20 g/L) was added to AA3 media (AA3Gly media) and DMSO was removed. From day 4 until the end of the differentiation, 5 µg/mL doxycycline was added to induce the three transcription factors. iPSCs used for differentiation were between passages 59 and 62, and the differentiation process was not interrupted by any freeze–thaw cycle.

#### Human liver microsomes (HLMs)

HLMs mixed gender, 150 donor pool, were purchased from BioIVT, US (product number X008070, lot: WGP).

#### Primary human hepatocytes (PHHs)

A mixed gender human hepatocyte pool from 10 donors were purchased from BioIVT, US (product number: X008001-P, lot: SWY) and handled according to manufacturer’s specifications. Cells were thawed using the OptiThaw Hepatocyte Medium (BioIVT) on Collagen I-coated plates. The cell density was 145.833 cells/cm^2^. Six hours after plating, media was changed from hepatocyte plating media to maintenance media. Cells were in culture no longer than 48 h.

### Immunofluorescence

HLCs differentiation was assessed by measuring five specific liver markers at days 22 and 44 of differentiation: CYP3A4, carboxylesterase 1 (CES1), albumin, alpha-1-antitrypsin and phosphoenolpyruvate carboxykinase (PEPCK) by immunofluorescence. Cell fixation and antibody staining were conducted as previously described (Meijer et al. 2024). The nucleus was stained with Hoechst 33342, and the secondary antibody for all three primary antibodies was Alexa 647 donkey-anti-rabbit IgG. The cells were imaged using a 20× water immersion objective on the Operetta CLS High-Content Imager (PerkinElmer) with confocal imaging, and image analysis was performed with the Harmony 4.9 software. List of primary and secondary antibodies can be found in Table S5.

### Glo assay to assess CYP3A4 activity

Glo assay (P450-Glo™ CYP3A4 Assay and Screening System, Promega, cat. V9001) is a luminescent method for measuring P450 CYP3A4 activity. CYP3A4 activity was measured in both mHepG2 and HLCs along the differentiation to evaluate at which stage HLCs and mHepG2 were metabolically active. Measurements were performed according to manufacturer’s indications, and data were normalized by protein amount (using the bicinchoninic acid (BCA) assay from Thermo Fisher, cat. 10678484).

### Gene expression analysis

Gene expression of CYP isoforms was measured using quantitative polymerase chain reaction (qPCR) in all hepatic models cultured in a 6-well plate format. RNA was isolated using RNeasy Mini Kit (Qiagen, cat. 74104) for HepaRG, HepG2, HLCs, and mHepG2, while a miRNeasy Mini Kit (Qiagen, cat. 217004) was used for PHHs. RNA from PHHs was isolated prior to plating to ensure the highest RNA quality. Complementary DNA (cDNA) was obtained using the iScript™ Reverse Transcription Supermix (Bio-Rad, cat. 1708841). A final cDNA concentration for all tested genes in the qPCR reaction was 0.2 ng/µL. Luna Universal qPCR Master Mix (BIOKE, cat. NEB M3003E) was used for the qPCR reactions. Each cDNA sample was measured in triplicate. Concentration and volume of reagents followed manufacturer’s specifications. Primers for *CYP1A1, CYP2A6, CYP2B6, CYP2C8, CYP2C9, CYP2C19, CYP2E1, CYP3A4,* and *CYP3A5* (see Table S12) were first tested in HepaRG, assuring an efficiency between 90 and 110%, while primers for *CYP1A2* and *CYP2D6* were tested in PHHs. Primer specificity was assessed by aligning the sequence to the human genome using BLAST. The same level of fluorescence threshold (ΔR) was kept across cell systems for each transcript (see Table S12). Analysis was performed in AriaDx Real-Time PCR (qPCR) Instrument from Agilent V.2.1. Results were expressed as relative quantification of the gene of interest to the housekeeping gene, in this case *GAPDH*, following Eq. (1):

1 $$\frac{{2}^{-{C}_{T\text{gene of intetest}}}}{{2}^{-{C}_{T\text{ GAPDH}}}}$$

To correct for inter-plate variation, a calibrator was used: cDNA of day 44 HLCs with a concentration of 0.03125 ng/µL amplified with *alpha*-*Tubulin* primers (keeping the Δ*R* set at 13 for all measurements). When the gene of interest and *GAPDH* were amplified in different plates, before applying Eq. (1), correction by the average ${2}^{-{C}_{T\text{average calibrator}}}$ of the plate calibrator was applied to each ${2}^{-{C}_{T\text{gene of interest}}}$.

### Protein quantification

Protein quantification of all cell models was performed using a targeted proteomics approach, as described elsewhere (Weiß et al. 2018). All cell models were cultured in 10 cm^2^ dish format, except for PHHs cultured for 48 h in 6-well plate format. Briefly, from each cell system, lysates were obtained and quantified for their total protein using the BCA assay (Thermo Fisher) prior analysis. Protein (150 μg) was proteolyzed with trypsin (Pierce Trypsin Protease, MS grade; Thermo Fisher) and surrogate peptides and internal standard peptides were precipitated with triple X proteomics (TXP) antibodies. Peptides were eluted and quantified using parallel reaction monitoring (PRM) (UltiMate 3000 RSLCnano and PRM-QExactive Plus from Thermo Fisher) (Belghit et al. 2021; Wuerger et al. 2022). Raw LC–MS data were processed using TraceFinder 4.1 (Thermo Fisher) and peptide amounts were calculated by the ratio between signal areas of endogenous peptides and isotopically labeled standards. Protein amounts were normalized and expressed as pmol/mg of extracted total protein. In case replicate measurements were below the limit of quantification (LOQ), ½ LOQ was used for further analysis instead, as previously described (Bailey and Michelson 2013).

### Microsomal incubations

Human liver microsomes (HLMs) were individually incubated with 10 known phase I and phase II metabolic probes, according to a modified protocol (Dekker et al. 2016), see Table 1. Concentrations of each metabolic probe were selected according to MS sensitivity, while maintaining cell viability in HepaRG. A resazurin assay was used to assess cell viability, as previously described (Jennings et al. 2007). The following concentrations of metabolic probes were used: bupropion (25 µM), phenacetin (30 µM), rosiglitazone (10 µM), diclofenac (75 µM), dextromethorphan (15 µM), chlorzoxazone (60 µM), midazolam (15 µM), benzydamine (15 µM), coumarin (250 µM) and 7-ethoxycoumarin (60 µM). Incubations were conducted containing 0.5 mg/mL HLMs in 100 mM potassium phosphate buffer, 5 mM MgCl_2_ and 2 mM EDTA pH = 7.4 (KPi buffer) with a NADPH regeneration system (NRS, 100 mM glucose-6-phosphate, 1 mM NADP^+^, and 5 U/mL glucose-6-phosphate dehydrogenase) in a final volume of 500 µL. Vials containing HLMs in KPi buffer with each drug were pre-incubated for 5 min at 37 °C under agitation in an Eppendorf Thermomixer R 1.5 mL Shaking Heater Block. The reaction was initiated by addition of NRS. Aliquots (50 µL) were taken at 15, 30, 60, 120 and 180 min and immediately quenched with 950 µL 80% (v/v) MeOH/water with internal standard, 2 µM meloxicam (80% MeOH + IS). As controls, HLMs were incubated with 0.2% (v/v) DMSO, and drug stability was assessed by parallel sampling; blank samples were taken, containing all, but HLMs.

**Table 1:** Metabolic probes used in this study to assign activity of specified xenobiotic isoenzymes (cytochrome P450s, CYPs; flavin monooxygenases, FMOs; and phase I and II enzymes), by following the formation of specific metabolites

Metabolic probe	Enzyme	Associated drug metabolite	Literature
Phenacetin	CYP1A2	Acetaminophen	(Kudo et al. 2000)
Coumarin	CYP2A6	7-Hydroxycoumarin	(Pitaro et al. 2022)
Bupropion	CYP2B6	1-Hydroxybupropion	(Costa et al. 2019)
Rosiglitazone	CYP2C8	*N*-Desmethylrosiglitazone/Hydroxyrosiglitazone	(Cox et al. 2000; Hruska et al. 2005)
Diclofenac	CYP2C9	4-Hydroxydiclofenac	(Poon et al. 2001; Boelsterli 2003)
Dextromethorphan	CYP2D6	Dextrorphan	(Taylor et al. 2016)
Chlorzoxazone	CYP2E1	6-Hydroxychlorzoxazone	(Quesnot et al. 2018)
Midazolam	CYP3A4	1-Hydroxymidazolam	(Nguyen et al. 2016)
Benzydamine	FMOs	Benzydamine *N*-oxide	(Fisher et al. 2002; Taniguchi-Takizawa et al. 2015)
7-Ethoxycoumarin	Phases I and II	7-Hydroxycoumarin, Coumarin-7-*O*-sulfate	(Feng et al. 2018)

### In vitro cell treatment

Cell systems HepG2, HepaRG, mHepG2 and HLCs were treated with the same 10 metabolic probes and equal concentrations, as mentioned for the microsomal incubations. Drug solutions were prepared in DMSO, with a final DMSO concentration of 0.2% (v/v) in all incubations. Six-well plates were used, and drugs were administered in media without FBS. Aliquots of exposed media (100 µL) were taken at 0.5, 1, 3, 6, and 24 h after administration (extracellular content) and immediately quenched in 80% MeOH + IS. Control samples (cells incubated with 0.2% (v/v) DMSO) and blank samples (drug stability without cells) were collected in parallel. For the intracellular content, after 24 h treatment (last time point), cells were washed with 0.9% (m/v) NaCl and immediately quenched with liquid nitrogen.

DNA quantification was measured from the remaining cell pellet, using Invitrogen™ Quant-iT™ PicoGreen™ dsDNA Assay Kit (Thermo Fisher, cat. P7589), following the manufacturer’s specifications. Total DNA was used for metabolome analysis normalization. This assay was preferred over total protein quantification to account for variation in extracellular matrix (ECM) content, cell debris and coating material, which greatly differed between cell models.

### Sample preparation for metabolomics analysis

Microsomal aliquots and cell extracellular contents previously quenched in 80% MeOH + IS were extracted for 30 min under agitation at 4 °C. Intracellular contents were extracted by scraping cells with 1.5 mL 80% MeOH + IS and incubated for 30 min under agitation at 4 °C. Both microsomal aliquots and intracellular contents were further centrifugated (15 min). All supernatants were freeze-dried and dissolved in 100 µL (microsomes and intracellular content) or 250 µL (extracellular content) of 50% (v/v) MeOH/water. A last centrifugation was performed (5 min), immediately before LC–MS analysis.

### LC–MS metabolomics analysis

All solvents used in metabolomics were LC–MS grade and ultrapure water was obtained from a water purification system (Milli-Q EQ 7000, Merck Life Science). The LC–MS system consisted of an ultra-high performance liquid chromatograph, UHPLC (Agilent 1290 UHPLC), coupled to a high-resolution time-of-flight mass spectrometer, MS (Agilent 6230B TOF), equipped with an electrospray ionization source (ESI). Chromatography was conducted using a reverse phase column (Waters XBridge BEH C18 2.1 mm internal diameter × 100 mm long, 2.5 µm particle size and 130 Å pore size) with a guard column (Waters XBridge BEH C18 3.9 mm × 5 mm, 3.5 µm particle size, 130 Å pore size) at 40 °C temperature and 0.2 mL/min flow rate. A linear gradient was applied: 10 to 80% of B (0.1% (v/v) formic acid in acetonitrile) in 18 min with a total run of 24 min (A, 0.1% (v/v) formic acid in ultrapure water). The following MS source parameters were used: nebulizer gas (nitrogen) at 20 psi, 325 and 400 °C of gas and sheath gas temperatures, with sheath gas flow of 12 L/min, using 4 kV and 3 kV of capillary voltage in positive and negative ion modes, respectively. The acquisition rate was 1 spectra/s and 1000 ms/spectrum in centroid mode. Mass calibration was done on-the-fly by infusing along the run a reference solution with masses 161.050873 and 922.009798 for positive ion mode while 112.985587 and 1033.988109 for negative ion mode. A volume of 1 µL was injected for each sample, and samples were kept at 10 °C before analysis. Acquisition was done under the control of MassHunter Workstation version 11.0 (Agilent). Data analysis, including signal integration, was done with MassHunter 10.0 (Agilent). Putative metabolite identification was proposed based on accurate mass (< 5 ppm) and relative retention time (based on analyte’s logP). Literature research was performed based on in vivo and in vitro studies conducted with the researched drugs (see Table S13). Initial metabolite identification was done in microsomal incubations, in which drug mass features were expected to decline along incubation, while drug metabolite mass features were expected to increase over time, be absent in control and absent or higher than in blank samples. The metabolic probes (drugs) in this study were annotated with a metabolite identification confidence level 1, since authentic standards were acquired together with all samples, while putative metabolites were annotated with a confidence level 2 (Malinowska and Viant 2019), with the exception of acetaminophen and 7-hydroxycoumarin, confirmed with authentic standards (see Table S14). Metabolite intensities in the extracellular content (at 24 h) were normalized by IS, and divided by the respective parent drug intensity in the blank samples (at 24 h), normalized by IS. When comparing different cell systems, these metabolite ratios were normalized by total DNA (in µg), using Eq. (2). The ratio of metabolite intensities to the parent compound in the blank sample accounts for potential drug degradation and matrix effects:

2 $$\text{Normalized Intensity}= \frac{\left(\frac{Metabolite Intensity in Media \left(24h\right)}{\text{Internal Standard }\left(\text{IS}\right)}\right)}{\frac{\text{Parent Compound Intensity in Blank }(24\text{h})}{\text{Internal Standard }\left(\text{IS}\right)}}\div \mu g \text{DNA}$$

### Statistical analyses

Statistical analyses were conducted in R, the statistical language, version 4.3 for processing and visualizing experimental data. Min–max scaling was employed as a normalization technique for metabolite intensities in the microsomal kinetics of drugs and their metabolites. Min–max scaling normalized metabolite intensity values between 0 and 1, crucial for standardizing measurements across samples and ensuring observed variability reflected biological rather than methodological differences (Choudhury et al. 2020). Summary statistics were computed, and results were displayed in multi-panel figures for each pharmaceutical compound. Protein expression analysis underwent slight increments to address low values and log2 transformations to stabilize variance for reliable ANOVA results. Significant expression differences were identified through post-hoc analysis using Tukey’s test (Choudhury et al. 2020). LC–MS metabolomics data were adjusted with a Box–Cox transformation optimized for ANOVA (Yu et al. 2022). Graphs were produced using GraphPad Prism 8.0.1.

## Results

This study aimed to characterize the drug metabolism machinery of the cell systems, mHepG2 and HLCs, obtained by an amino acid-rich media regimen, using an integrated multi-omics approach. HLCs were tested at two stages of differentiation, days 22 and 44, to assess metabolic capacity at two time points of differentiation. Besides CYP3A4, all tested hepatic markers (carboxylesterase 1, albumin, alpha-1-antitrypsin, and phosphoenolpyruvate carboxykinase) were present in both stages of the differentiation, Fig. S2. These markers indicated that at day 22, HLCs already exhibited hepatocyte-like characteristics and morphology, as evident by confocal imaging, Fig. S3. Noticeable differences in cell morphology were observed between HepG2 and mHepG2, and between day 22 HLCs and day 44 HLCs. Like PHHs, both HepaRG and day 44 HLCs presented polynuclear cells, a feature commonly observed in mature hepatocytes, Fig. S3.

### Matured hepatocyte-like cells express drug-metabolizing CYP transcripts

**Fig. 1:**
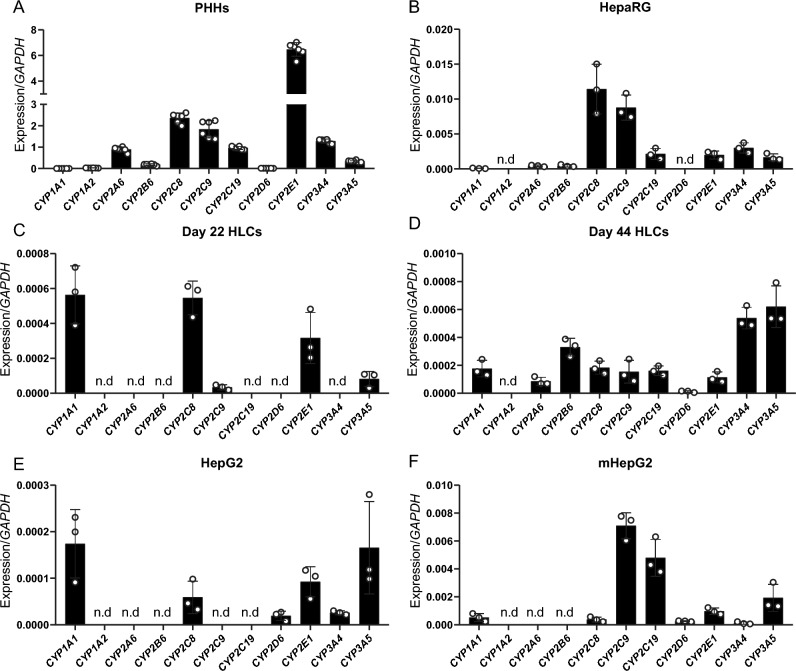
Expression of drug-metabolizing CYPs differs across hepatic cell systems. Gene expression obtained by qPCR, relative to expression of housekeeping gene *GAPDH* for CYP isoenzymes family 1: *CYP1A1* and *CYP1A2*, family 2: *CYP2A6, CYP2B6, CYP2D6, CYP2C8, CYP2C9, CYP2C19* and *CYP2E1*, and family 3: *CYP3A4, CYP3A5*. Cell models tested were primary human hepatocytes (**A**, PHHs); **B** HepaRG; iPSC-derived HLCs, following a differentiation protocol for 22 days (**C**, day 22 HLCs) and for 44 days (**D**, day 44 HLCs); **E** HepG2; and metabolically active HepG2 (**F**, mHepG2). Results are represented as average ± SD; n.d. = not detected. *N* = 3 replicates from the same cell line or batch of differentiation (3 independent RNA isolated wells). Only for PHHs: *N* = 6 replicates from the same RNA isolation of a mixed donor pool cryopreserved vial

To characterize phase I metabolism in human hepatic cell models, the transcript levels of 11 isoforms among the 57 human CYP isoenzymes were assessed, Fig. 1. The selection of isoforms was based on their substrate specificity and tissue site (Guengerich 2005). These isoforms are known to be expressed in the liver with established roles in the metabolism of many pharmaceutical drugs (Guengerich 2021).

Expression was first tested in PHHs, expected to have high coverage and activity, Fig. 1A. CYP2E and CYP2C families showed the highest expression, followed by CYP3A and CYP2A families. CYP1A family is known for its reduced expression, being *CYP1A1* the isoform with the lowest levels, from all tested isoforms. *CYP1A1* is also expressed extra-hepatically, being the least liver-specific CYP among the tested isoforms; explaining the low expression found in PHHs (Guengerich 2005; Lang et al. 2019). HepaRG showed CYP expression for all but 2 isoforms: *CYP1A2* and *CYP2D6*, Fig. 1B. Isoforms *CYP2C8* and *CYP2C9* were particularly high. iPSCs were differentiated using the protocol of Boon et al. 2020, and assessed at two different time points (day 22 and day 44). Transcripts for *CYP2A6*, *CYP2B6*, *CYP2D6*, *CYP2C19*, and *CYP3A4* were found to be expressed in HLCs after at least 40 days of differentiation, Fig. 1C, D. On day 44, HLCs express all tested CYP isoforms, except for *CYP1A2*, that was only expressed in PHHs. Metabolically active HepG2 (mHepG2), previously studied by Boon et al. 2020, are HepG2 cells submitted to a differentiation protocol with enriched levels of amino acids. Levels of all CYP transcripts expressed in HepG2 were increased in mHepG2, and even some isoforms that were not present in HepG2 cells were expressed in mHepG2 cells (*CYP2C9* and *CYP2C19*) Fig. 1E, F. Besides PHHs, qualitatively, day 44 HLCs exhibit the highest CYP transcript coverage from all the tested cell models, only missing *CYP1A2* expression.

It should be noted that CYP transcript levels in all tested cell systems were lower compared to PHHs. However, it is important to consider that RNA isolation from PHHs was performed prior to plating—directly from the cryopreserved vial—thus resulting in an overrepresentation of the expression compared to cultured PHHs, which tend to de-differentiate over time. For day 44 differentiated HLCs, most isoform transcript levels were lower than in HepaRG. Levels of CYP transcripts in mHepG2 appeared comparable to those in HepaRG. Of particular significance in drug metabolism is the isoform CYP3A4, known for its broad substrate specificity. *CYP3A4* expression levels were highest in HepaRG, followed by levels in day 44 HLCs; the latter being 20 times higher than in HepG2 and 4 times higher than in mHepG2. Interestingly, expression of *CYP2D6*, known for its role in the metabolism of anti-psychotics, appeared highest in mHepG2 (not expressed in HepaRG, and low expression in HepG2 and day 44 HLCs).

### Metabolically active HepG2 accumulate multiple CYP isoenzymes

**Fig. 2:**
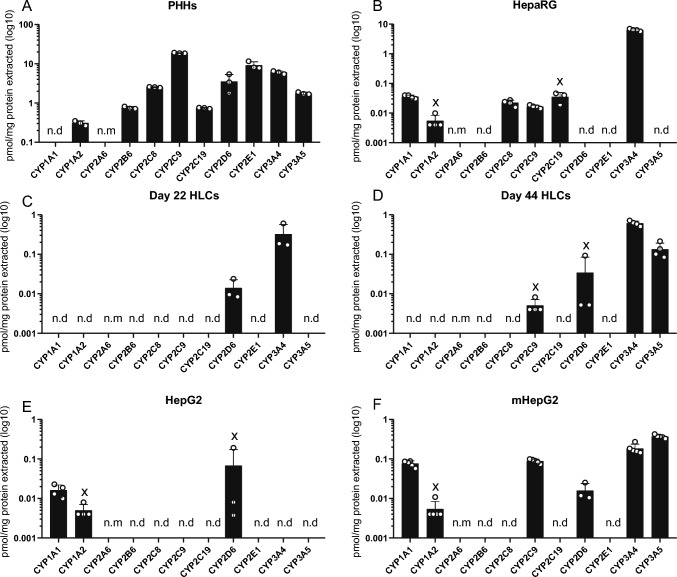
Protein levels of drug-metabolizing CYPs differ across hepatic cell systems. Protein quantification (pmol of protein/mg total protein) obtained by targeted proteomics for CYP isoenzymes family 1: CYP1A1 and CYP1A2, family 2: CYP2A6, CYP2B6, CYP2D6, CYP2C8, CYP2C9, CYP2C19 and CYP2E1, and family 3: CYP3A4, CYP3A5, on primary human hepatocytes (**A**, PHHs); **B** HepaRG; iPSC-derived HLCs, following a differentiation protocol for 22 days (**C**, day 22 HLCs) and for 44 days (**D**, day 44 HLCs); **E** HepG2; and metabolically active HepG2 (**F**, mHepG2) (average log10 ± SD); n.d. = not detected; n.m. = not measured. *N* = 3–5 replicates from the same cell model or batch of differentiation (samples prepared as independent lysates). In case some, but not all biological replicates were below the quantification limit, (x), ½ limit of quantification was used for the analysis (Bailey and Michelson)

Targeted proteomics analysis quantified basal levels of CYP isoenzymes in different hepatic cell systems (Fig. 2). The same CYP isoforms were tested at the protein level, as the ones tested at the transcript level (Fig. 1), except for CYP2A6 which was not included in the proteome panel. As for the transcript quantification, CYP expression levels in PHHs were generally higher compared to all hepatic cell systems tested. However, protein levels did not correlate to transcript levels. In PHHs for instance, the high transcript levels of *CYP2E1* (Fig. 1A, compared to other isoforms) were not observed at the protein level; instead, levels of CYP2E1 protein were comparable to those quantified for the isoenzymes CYP3A4 or CYP2C9, Fig. 2A. CYP1A1 protein was not detected in PHHs. Furthermore, protein levels in the tested cell systems were here found to be comparable to PHHs (Fig. 2), unlike transcript levels (Fig. 1). This can be the result of differences in culture conditions (to note: protein determination was performed in 48 h cultured PHHs). In HepaRG, CYP3A4 was the highest quantified CYP protein, Fig. 2B, and levels were comparable to those of PHHs. In addition, isoenzymes CYP1A1, 1A2, 2C8, 2C9 and 2C19 were quantified in this cell system. Whereas only 2 CYP enzymes, CYP2D6 and CYP3A4, were detected in day 22 HLCs, on day 44 of differentiation, 2 additional isoforms were quantified, CYP2C9 and CYP3A5, Fig. 2C, D. Although transcripts for most CYPs were detected in day 44 HLCs, Fig. 1D, they were either not translated into protein or protein levels remained below the level of quantification, Fig. 2D. Protein levels of CYP3A4 doubled in 22 days (from 0.3 to 0.6 pmol/mg extracted protein). mHepG2 showed remarkable accumulation of CYP proteins. ‘Standard’ proliferating HepG2 cells are notorious for low levels of CYP3A4 (Aninat et al. 2006; Chen et al. 2021), and indeed only isoenzymes CYP1A1, 1A2 and 2D6 were quantified, Fig. 2E. By contrast, culturing HepG2 in high amino acid medium resulted in the detection of 3 other CYP isoforms at the protein level: 2C9, 3A4 and 3A5, Fig. 2F. Protein levels of CYP3A4 were however lower than those found in day 44 HLCs (0.18 compared to 0.6 pmol/mg, respectively), in line with findings at the transcript level.

Certain discrepancies were encountered between protein and transcript levels, likely due to different detection sensitivities in the used techniques or antibody selectivity used in our targeted proteomic panel. At the transcript level, many more isoforms were quantified, in particular in HLCs, not detected at protein level. Surprisingly, CYP1A2 protein levels were detected in HepaRG, HepG2, and mHepG2, while the transcript remained not detected. CYP2D6 and 3A4 proteins were quantified in day 22 of differentiation in HLCs, while the corresponding transcripts were not detected. Like in transcript expression, more proteins (e.g., CYP2C9, CYP3A5, CYP3A4) were detected in day 44 HLCs and mHepG2 compared to more immature models: day 22 HLCs and HepG2, respectively. Nevertheless, at a protein level, besides PHHs, mHepG2 and HepaRG have a wider CYP protein coverage compared to the remaining tested in vitro models.

### Model drugs undergo CYP-mediated metabolism in human liver microsomes

To assess drug metabolic capacity in HLCs and mHepG2, HLMs were first used to establish our methodology for studying all possible CYP-mediated metabolism. These cellular fractions of the endoplasmic reticulum, microsomes, contain many phase I enzymes, including CYPs, responsible for drug biotransformation. While HLMs do not reflect the whole cellular milieu, being deprived of important phase II enzymes, such as SULTs or GSTs (Sun et al. 2024), they were essential to validate our LC–MS metabolomics approach.

For this purpose, 10 pharmaceutical drugs, Table 1, were subjected to independent incubation with HLMs. These drugs were chosen for their well-known CYP-isoenzyme-specific biotransformation. For example, phenacetin is known to be de-ethylated to acetaminophen by CYP1A2. While certain metabolic reactions may be mediated by several CYP isoenzymes, the list of proposed metabolic probes is generally associated with a certain CYP isoenzyme activity. Time series incubations (*t* = 15, 30, 60, 120 and 180 min) were conducted and analyzed by LC–MS-based metabolomics, using both positive and negative ion modes, aiming for the widest metabolite coverage. To assist in our metabolite identification, extensive literature research was performed to list all possible reported metabolites of each drug (see Table S13). Theoretical masses of molecular ions ([M + H]^+^ in positive mode or [M-H]^−^ in negative mode) of reported metabolites were used to interrogate the experimental dataset. As expected, given the physico-chemical properties of pharmaceutical drugs, most metabolites were found in positive ion mode. Analytical features of each metabolite are listed in Table S14. A common biotransformation mediated by CYPs is hydroxylation. Most of the here reported hydroxylated metabolites remain of unknown position, relying only on accurate mass and literature for their putative identification, as commercial standards were often absent. Likewise, for other reported metabolites, their putative functional group position(s) remained unassigned.

**Fig. 3:**
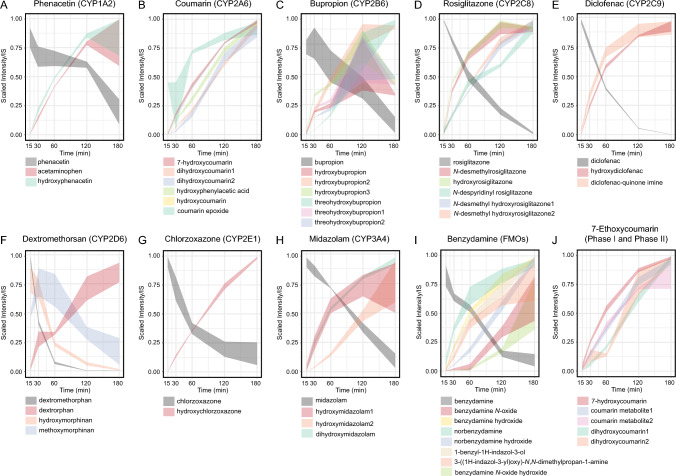
Kinetics of phase I metabolism of model drugs in human liver microsomes (HLMs). Model drugs were used as substrates in enzyme (isoform)-specific metabolic reactions, forming a marker metabolite: **A** phenacetin to acetaminophen (CYP1A2); **B** coumarin to 7-hydroxycoumarin (CYP2A6); **C** bupropion to hydroxybupropion (CYP2B6); **D** rosiglitazone to *N*-desmethylrosiglitazone (CYP2C8); **E** diclofenac to hydroxydiclofenac (CYP2C9); **F** dextromethorphan to dextrorphan (CYP2D6); **G** chlorzoxazone to hydroxychlorzoxazone (CYP2E1); **H** midazolam to hydroxymidazolam (CYP3A4); **I** benzydamine to benzydamine-*N*-oxide (FMOs); **J** 7-ethoxycoumarin to 7-hydroxycoumarin (phase I and phase II metabolism). Drugs (in black), marker metabolites (in red) and other identified metabolites (multiple colors) were analyzed over five-time points: 15, 30, 60, 120 and 180 min using LC–MS metabolomics, in either positive or negative ion mode. Drug and assigned putative metabolite LC–MS intensities were normalized using min–max scaling (average ± SD, indicated by the width of the line). *N* = 3 replicates, independent incubations per drug. The drugs coumarin (**B**) and 7-ethoxycoumarin (**J**) showed poor LC–MS ionization and thus were not displayed

Many putatively identified metabolites were detected for the 10 incubated metabolic probes and corresponding kinetic curves in HLMs were obtained, Fig. 3. As expected, intensity of the drug (parent compound) decreased over time, while the abundance of the respective metabolites increased within the same time frame. Phenacetin led to the metabolite acetaminophen indicating CYP1A2 activity, Fig. 3A. 7-Hydroxycoumarin was one of the metabolites obtained from the metabolism of coumarin, demonstrating CYP2A6 activity, Fig. 3B, even if the parent compound itself, coumarin, was not consistently detected, probably due to matrix effects. Other metabolites of coumarin were also detected, including the reactive metabolite coumarin epoxide. CYP2B6 activity was assessed by the production of hydroxybupropion from bupropion. Actually, several hydroxylated species of bupropion were detected, Fig. 3C. Rosiglitazone was used as a probe for CYP2C8/9 activity. The marker metabolite *N*-desmethylrosiglitazone was produced, confirming the activity of this isoenzyme, Fig. 3D. Diclofenac was used to confirm CYP2C9 activity, leading to the formation of 4-hydroxydiclofenac, Fig. 3E. Isoform CYP2D6 was assessed by measuring dextrorphan, a metabolite of dextromethorphan, Fig. 3F. Chlorzoxazone was used to test CYP2E1 activity, leading to the detection of the putative metabolite 6-hydroxychlorzoxazone, Fig. 3G. Midazolam was also incubated in HLMs as a marker of CYP3A4 activity, by the formation of 1-hydroxymidazolam, Fig. 3H. With less abundance, midazolam was also metabolized through CYP3A4 to 4-hydroxymidazolam (here called hydroxymidazolam2), Fig. 3H**.** Flavin monooxygenase (FMO) activity was tested by detecting the metabolite benzydamine-*N*-oxide in HLMs incubated with benzydamine, Fig. 3I. 7-Ethoxycoumarin was used as a probe for phase I and phase II activity. As expected, conjugated metabolites were not found in HLMs incubated samples, but the phase I metabolite 7-hydroxycoumarin was detected, Fig. 3J, even if, like with coumarin, 7-ethoxycoumarin could not be detected.

### Metabolically active HepG2 exhibits improved phase I metabolism activity

**Fig. 4:**
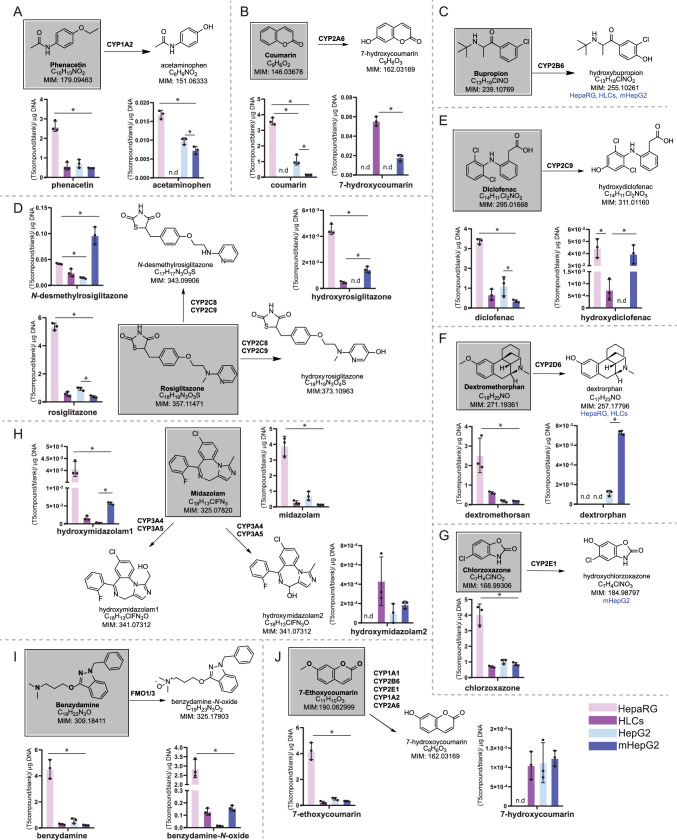
Phase I metabolic activity in the human hepatic cell systems HepaRG, HLCs, HepG2 and mHepG2. Model drugs (structures in grey box) were used as substrates in enzyme (isoform)-specific metabolic reactions, forming a marker metabolite (with structure): **A** phenacetin to acetaminophen (CYP1A2); **B** coumarin to 7-hydroxycoumarin (CYP2A6); **C** bupropion to hydroxybupropion (CYP2B6); **D** rosiglitazone to *N*-desmethylrosiglitazone (CYP2C8); **E** diclofenac to hydroxydiclofenac (CYP2C9); **F** dextromethorphan to dextrorphan (CYP2D6); **G** chlorzoxazone to hydroxychlorzoxazone (CYP2E1); **H** midazolam to hydroxymidazolam (CYP3A4); **I** benzydamine to benzydamine-*N*-oxide (FMOs); **J** 7-ethoxycoumarin to 7-hydroxycoumarin (phase I and phase II metabolism). Drugs (in a box) and marker metabolites were analyzed in extracellular contents after 24 h using LC–MS metabolomics. Metabolite intensities in HepaRG (pink), HLCs (purple), HepG2 (light blue), and mHepG2 (dark blue) were normalized to drug intensity in the blank and to total DNA (average ± SD, *p < 0.05). *N* = 3 replicates per cell model (from the same cell line or batch of differentiation) and drug (3 independent extracted wells). In case metabolites were only found intracellularly in a certain cell system, the name of the cell system was added below the drug structure. Bupropion (**C**) showed poor LC–MS ionization, and thus was not displayed. n.d. = not detected

Characterizing the drug metabolism of model drugs, Table 1, using HLMs, in combination with our LC–MS-based xenobiotic metabolomics approach, allowed obtaining the appropriate setup for studying the xenobiotic capacity in hepatic in vitro systems. Table 1 drugs are used to challenge HepG2, HepaRG, mHepG2 and day 44 HLCs for their drug metabolism. As the presence of CYP transcripts and proteins were limited in day 22 HLCs (Fig. 1C and 2C), in particular CYP3A4, this cell system was excluded from further metabolic studies (see Fig. S4). Intracellular and extracellular drug disappearance and metabolite formation was monitored using LC-ESI^−/+^-MS. This approach allowed monitoring numerous metabolites, beyond the described CYP metabolite markers, Table S14. All metabolites here described were putatively identified, except acetaminophen and 7-hydroxycoumarin for which authentic standards were used for metabolite confirmation, Fig. 4.

CYP1A2 activity was detected in all cell systems, except for HLCs, by the presence of acetaminophen in the culture medium, Fig. 4A. This result is in line with the fact that neither CYP1A2 transcript (Fig. 1D) nor protein (Fig. 2D) was detected in this cell system. No CYP2A6 activity was observed in HepG2 cells, but was present in mHepG2 as well as in day 44 HLCs, showing higher metabolic activity in the latter through the formation of 7-hydroxycoumarin, Fig. 4B. CYP2B6 activity was identified in all cell systems, except for HepG2; however, due to the poor ionization of bupropion, hydroxybupropion was reported only qualitatively (Fig. 4C). CYP2C8/9 activity was higher in both mHepG2 and HepaRG, Fig. 4D, E. CYP2C9-produced metabolite was not found in HepG2, indicating lack of activity in this cell line, Fig. 4E. The marker metabolite of CYP2D6 was found in all cell systems (in HepaRG and HLCs only intracellularly), Fig. 4F, even if there was an absence of CYP2D6 transcript (Fig. 1B) or protein (Fig. 2B) detected in HepaRG. In this cell model, it is plausible that metabolic activity is rather conducted through another CYP isoform, a phenomenon known as enzyme promiscuity (Becker et al. 2021) or lately classified as permissiveness (Hansen et al. 2021), documented for the CYP superfamily. CYP2E1 activity was only identified in mHepG2 (hydroxychlorzoxazone was only found intracellularly), Fig. 4G. The CYP isoforms most actively studied, CYP3A4/5, here tested using midazolam, showed metabolic activity in all cell systems, being HepG2 the cell line with the poorest activity, seen from the lowest formation of the marker metabolite 1-hydroxymidazolam; however, it did generate comparable levels of 4-hydroxymidazolam (here called hydroxymidazolam2), a less abundant metabolite of midazolam (Fig. 4H). FMO activity was assessed by the production of benzydamine-*N*-oxide, using benzydamine as a substrate. In addition, in this case, HepG2 showed the poorest activity, Fig. 4I. The metabolic reaction forming 7-hydroxycoumarin from 7-ethoxycoumarin can be mediated by several CYP isoforms, so it was used as a proxy for general phase I metabolism activity. All cell systems could metabolize this substrate, except HepaRG, evident from the lowest consumption of 7-ethoxycoumarin, Fig. 4J.

From this metabolic analysis, the only cell model tested able to show all assessed enzymatic activities was mHepG2. HLCs displayed metabolic activity for most CYP isoforms tested, except for CYP1A2 and CYP2E1. HepaRG, generally considered a preferred cell model over HepG2 in drug metabolism studies, showed lack of CYP2A6 and CYP2E1. Accordingly, the least performing cell line in studying drug metabolism was HepG2, lacking CYP2A6, CYP2B6, CYP2C9, and CYP2E1 activities.

**Fig. 5:**
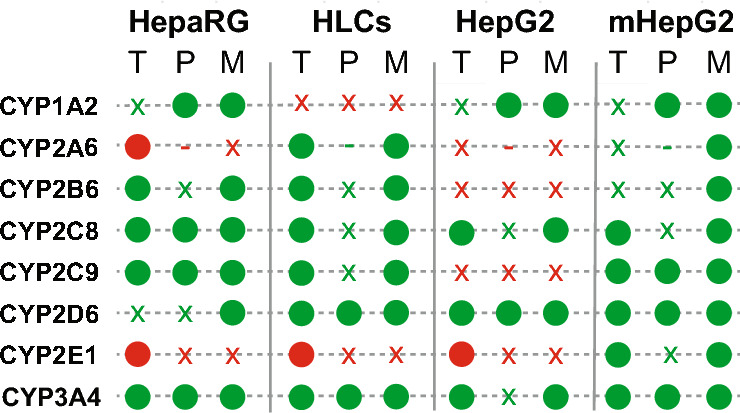
Qualitative multi-omics representation of drug-metabolizing CYP transcriptome (T), proteome (P), and metabolome (M) in human hepatic cell systems HepaRG, HLCs, HepG2 and mHepG2. The phase I CYP isoforms CYP1A2, CYP2A6, CYP2B6, CYP2C8, CYP2C9, CYP2D6, CYP2E1, and CYP3A4 were detected (o) or not detected (x) across the three omics, leading to the presence (in green) or absence (in red) of metabolic activity in the respective cell systems. CYP2A6 was not measured at the protein level (−)

Phase I characterization in hepatic models was integrated at the transcript, protein, and metabolic levels, Fig. 5. Although day 44 HLCs demonstrated the widest CYP expression at the transcript level, mHepG2 exhibited broader protein expression and metabolic activity. Based on these findings, day 44 HLCs were inadequate for investigating drug metabolism via CYP1A2 and CYP2E1; the HepaRG model was not suitable for studying drug metabolism mediated by CYP2A6, CYP2D6, and CYP2E1; and HepG2 lacked CYP2A6, CYP2B6, CYP2C9, and CYP2E1 activities.

### Day 44 HLCs and mHepG2 exhibit phase II metabolism activity

**Fig. 6:**
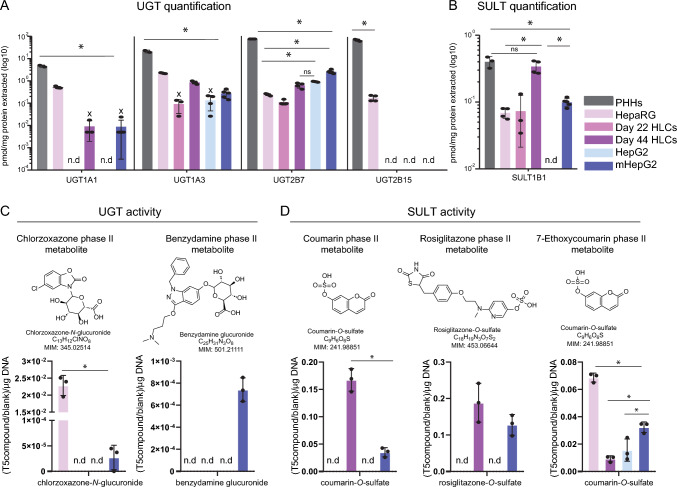
Phase II metabolism in the human hepatic cell systems HepaRG, HLCs, HepG2 and mHepG2. Protein quantification of 4 UGT isoenzymes (**A**) and 1 SULT isoenzyme: SULT1A1 (**B**). Protein levels in PHHs, HepaRG, day 22 HLCs and 44, mHepG2, and HepG2 were determined by targeted proteomics (average log10 (pmol of protein/mg protein extracted) ± SD, *p < 0.05, ns = not significant); n.d. = not detected. *N* = 2–5 replicates from the same cell model or batch of differentiation (samples prepared as independent lysates). In case some, but not all replicates were below the quantification limit (x), ½ limit of quantification was used for representation (Bailey and Michelson); UGT2B7 in PHHs was above quantification limit; therefore, this value was illustrated alternatively. UGT (**C**) and SULT (**D**) metabolic activity. Phase II metabolites of model drugs chlorzoxazone, benzydamine, coumarin, rosiglitazone and 7-ethoxycoumarin analyzed in extracellular contents of each cell system, after 24 h using LC–MS metabolomics. Metabolite intensities in HepaRG (pink), HLCs (purple), HepG2 (light blue), and mHepG2 (dark blue) were normalized to drug intensity in the blank and to total DNA (average ± SD, *p < 0.05). *N* = 3 replicates per cell model (from the same cell line or batch of differentiation) and drug (3 independent extracted wells)

Two superfamilies of enzymes accounting for the majority of phase II metabolism in humans are UGTs and SULTs (Runge-Morris and Kocarek 2009). To characterize them, targeted proteomics was used to quantify four UGT isoforms (UGT1A1, UGT1A3, UGT2B7, and UGT2B15) and one SULT isoform (SULT1B1), in the five hepatic cell systems HepG2, HepaRG, mHepG2, and HLCs on day 22 and on day 44. PHHs cultured for 48 h were used as a positive control, having the highest abundance of tested proteins, Fig. 6A, B. UGT1A1 protein was quantified in HepaRG, mHepG2, and HLCs, while UGT2B15 was only quantified in HepaRG. UGT1A3 and UGT2B7 protein was found in all cell systems, Fig. 6A. The isoform 1B1 of SULT was present in all cell models, except for HepG2, Fig. 6B. SULT1B1 demonstrated similar levels in day 44 HLCs, when compared to PHHs. In line with phase I metabolism, the day 44 HLCs and mHepG2 showed higher phase II protein levels compared to their respective less mature cell systems, day 22 HLCs and HepG2.

To study phase II metabolism in the tested cell systems, LC–MS-based metabolomics identified phase II metabolites, using model drugs (Fig. 6, Table 1). Given the hydrophilicity of phase II conjugates, most phase II metabolites were detected in negative ion mode LC–MS, Table S14. The metabolic fate of benzydamine and chlorzoxazone led to the identification of glucuronides in mHepG2 and in HepaRG (benzydamine glucuronide and chlorzoxazone glucuronide), Fig. 6C. Sulfated conjugates were detected in incubations with coumarin, rosiglitazone and 7-ethoxycoumarin, Fig. 6D. Consistent with the proteomics results, HLCs presented higher SULT activity, evident from the higher levels and coverage of sulfate metabolites (rosiglitazone-*O*-sulfate, and coumarin-*O*-sulfate), compared to the remaining cell models. From the tested drugs, HepG2 could only produce the phase II metabolite coumarin-*O*-sulfate from 7-ethoxycoumarin.

### LC–MS-based metabolomics workflow allows for a comprehensive overview of drug metabolism

**Fig. 7:**
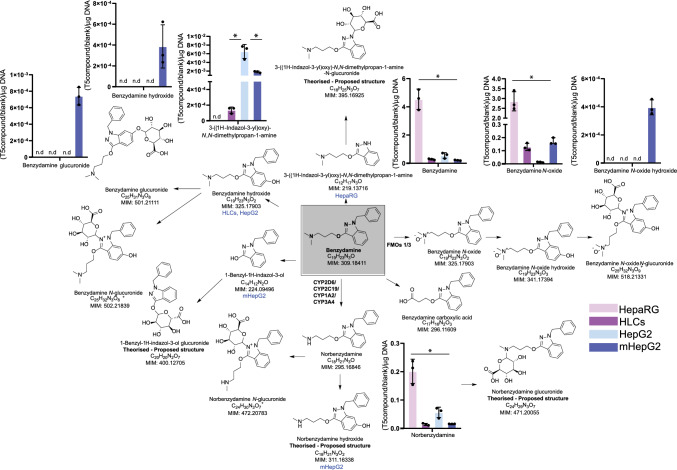
Drug metabolism map of benzydamine. Metabolic fate of benzydamine (in grey box) with assigned metabolic enzymes and (putative) metabolites found in the literature and experimentally (in this study). LC–MS metabolite intensities of extracellular contents in HepaRG (pink), HLCs (purple), HepG2 (light blue), and mHepG2 (dark blue) were normalized to drug intensity in the blank and to total DNA (average ± SD, *p < 0.05). *N* = 3 replicates per cell model (from the same cell line or batch of differentiation) and incubation condition (3 independent extracted wells). In case metabolites were only found intracellularly in a certain cell system, the name of the cell system was added below the drug structure; n.d. = not detected; “theorized-proposed structure” indicates metabolites not reported in the literature, but of likely occurrence through phase I and phase II metabolism

The established LC–MS-based metabolomics approach allowed for the simultaneous characterization of many putative metabolites produced by phase I and II metabolism of model drugs, across various cell systems, extracellularly and intracellularly. This approach was here exemplified in the characterization of benzydamine drug metabolism, Fig. 7. According to the literature, first metabolic step of this anti-inflammatory drug in the liver is conducted by FMO isoforms 1 and 3. Moreover, benzydamine undergoes other phase I (through CYP3A4, CYP2C19, CYP2D6 and CYP1A2) and phase II reactions (glucuronidation) (Fisher et al. 2002; Taniguchi-Takizawa et al. 2015). From our experimental data, at the qualitative level, mHepG2 showed the highest metabolite coverage in benzydamine’s metabolic map. In this cell model, 8 benzydamine metabolites were detected, including a glucuronide (benzydamine glucuronide). HepaRG exhibited a lower number of metabolites detected, benzydamine-*N*-oxide and norbenzydamine (3-((1H-indazol-3-yl)oxy)-*N,N*-demethylpropan-1-amine only intracellularly). However, higher levels of these 2 metabolites were found in HepaRG compared to other models, Fig. 7. These results highlight the value of HepaRG in studying CYP3A4-mediated metabolism, but it remains a limited cell model in studying drug metabolite formations at large.

Using our LC–MS metabolomics workflow to study the metabolic fate of 10 model drugs (Table 1) in the various cell systems, a total of 32 metabolites were identified using the mHepG2 model, making it the in vitro system with the highest metabolite coverage. This was followed by 23 metabolites in HLCs, 20 in HepaRG, and 17 in HepG2 (see Table S14). Furthermore, reactive metabolites such as coumarin epoxide (found in HLMs) and diclofenac quinone imine (in HLMs, HepaRG and mHepG2) were identified with this untargeted approach. The identification of toxic intermediates, products of drug metabolism, is an essential milestone for the prevention of DILI and toxic adverse effects.

## Discussion

With the development of new stem cell technologies, in vitro systems are becoming ever performing, gathering a myriad of attractive advantages. Among these applications are: disease modeling (by studying metabolic disorders such as non-alcoholic fatty liver disease, hepatitis, and liver fibrosis under controlled conditions), drug metabolism and toxicity (by providing a platform for screening drug metabolism, assessing hepatotoxicity, and predicting adverse drug reactions before clinical trials) and personalized medicine (by enabling patient-specific drug testing and metabolic studies, reducing the risk of unpredictable responses in individuals) (Hannoun et al. 2016; Kozyra et al. 2018; Li et al. 2019; Aboul-Soud et al. 2021; Ghosh et al. 2021; Mukhopadhyay et al. 2024). The tractability and scalability of iPSCs derived models is attractive, yet they remain poorly characterized, in particular for use in metabolism studies. Our study used a multi-omics approach to characterize two emerging cell systems: iPSC-derived HLCs and mHepG2 for their drug metabolism capacity and benchmark them against established in vitro cell systems, HepG2 and HepaRG, and the gold standards in drug metabolism studies, HLMs and PHHs. Eleven CYP isoforms and some phase II enzymes were characterized, essential in the metabolism of a variety of xenobiotics including pharmaceutical drugs.

To this end, HepG2 and HepaRG were utilized in this study as established cell lines, to provide a frame of reference. Even though HepG2 and HepaRG are extensively studied cell models, information about CYP expression remains fragmented and outdated. The large variability in cell culture, differentiation, and induction protocols in HepG2 and HepaRG, makes the interpretation of reported literature a challenging task (Stanley and Wolf 2022). HepG2 is known to have a low basal expression of CYPs (Aninat et al. 2006; Chen et al. 2021). Consequently, only a limited number of drug metabolism studies have utilized this cell line in basal conditions, without CYP inducers or genetic modifications (Choi et al. 2015; Boon et al. 2020; Chen et al. 2021). Chen and colleagues characterized HepG2 drug metabolism at basal and CYP-genetically overexpressed conditions (Chen et al. 2021). They reported basal HepG2 expression levels of isoforms *CYP1A1, CYP2E1* and *CYP3E5*, while *CYP1A2* remained under the detection limit, in line with our study. *CYP2C8*, *CYP3A5,* and *CYP2D6* were found not to be expressed, while *CYP2A6* and *CYP2B6* were present, opposite to here reported. Moreover, Chen and colleagues did not detect any CYP isoforms at basal HepG2 protein level by Western blot, and there was not any metabolic activity observed for CYP1A2, CYP3A4 nor CYP2D6, as measured by LC–MS. This dissonance with our study may suggest variation in experimental conditions and methods applied (Chen et al. 2021; Stanley and Wolf 2022). The same authors characterized the HepaRG CYP transcriptome. Consistent with our findings, *CYP2D6* was not expressed in this cell line, and *CYP1A2* showed very low expression (Chen et al. 2021). Despite these observations, cell lines such as HepG2 and HepaRG offer an attractive tractability for being expandable, and relatively easy to handle. In certain studies, they may be utilized as an initial cellular background from which hypotheses can be generated and expanded in other more physiologically relevant systems.

In studying phase I metabolism, specifically CYP metabolism, HLMs remain a great tool, as demonstrated in this study (Sun et al. 2024). HLMs were an essential resource for the development of our LC–MS-based metabolomics workflow to study xenobiotic metabolism. Biokinetics studies were easily conducted in HLMs, even allowing monitoring toxic intermediates. Due to their high concentration of CYP enzymes and shorter incubation times, HLMs are efficient in the identification of many metabolic intermediates, when combined with LC–MS metabolomics. However, HLMs remain a perishable resource obtained from liver biopsies and do not contain cytosolic drug metabolic activity, which excludes various phase II enzymes (Sun et al. 2024). For a comprehensive hepatic drug metabolism, PHHs can be used instead, but they also gather a major limitation of being a perishable resource and rapidly de-differentiating, resulting in loss of metabolic activity (Boon et al. 2020; Ghosh et al. 2021).

For this reason, the characterization of promising iPSC-derived hepatocytes, as well as mHepG2 was comprehensively conducted for their drug metabolism in this study. Both systems were obtained from making use of high amino acid concentration in their media (Boon et al. 2020). mHepG2 is perhaps the most surprising cell system by having the exact same genetic background as HepG2, but through the applied differentiation protocol (Boon et al. 2020) led to expression and activity on 8 different CYP isoforms: CYP1A2, 2A6, 2B6, 2C8, 2C9, 2D6, 2E1 and 3A4. mHepG2 is consequently an in vitro system of high relevance in studying drug metabolism. mHepG2 not only improved the expression and functionality of phase I but also phase II enzymes UGTs and SULTs. This integrated study led to the proposal that this cell system is the most performant in addressing the metabolism of a variety of drug chemistries that may use different CYP isoenzymes.

HLCs showed metabolic activity in 6 CYP isoforms: CYP2A6, 2B6, 2C8, 2C9, 2D6, and 3A4. HLCs demonstrated a high metabolic activity comparable to the activities found in mHepG2 and HepaRG, while having lower transcript and protein levels of phase I enzymes. A possible explanation for this phenomenon may be enzyme activation. CYPs are known to be induced by various substrates. Beta-naphthoflavone, phenobarbital, and rifampicin are known CYP inducers, leading to transcript and functional differences (Gerets et al. 2012). Gerets and colleagues found that CYP transcript and functional chemical-induction differed between cell models, specifically HepG2 and HepaRG (Gerets et al. 2012). A > 40-fold increase in CYP3A4 and CYP1A2 activity was obtained in HepaRG when these isoforms were chemically induced; in comparison, induction was much less effective in HepG2 (Gerets et al. 2012). CYP1A2 activity was reported in HLCs in a previous study using human embryonic stem cells, suggesting that lack of CYP1A2 in our HLCs may be a consequence of the iPSCs’ donor-specific traits (Boon et al. 2020). Actually, Ghosh and colleagues reported that iPSCs derived HLCs-CYP3A4 activity was donor-dependent (Ghosh et al. 2021). Thus, iPSCs may retain specific donor-expression or activity of certain enzymes, in line with human inter-individual variability. Furthermore, the presence of phase II metabolic activity in HLCs was reported here, supported by proteome and metabolome data. These findings are corroborated by gene expression data reported previously (Ek et al. 2007; Si-Tayeb et al. 2010). Phase II metabolism, much less studied, is of extreme importance in promoting drug clearance, and may even be object of production of toxic intermediates (e.g., acyl-glucuronides) (Iwamura et al. 2017).

A recent comparison between HLCs (following a 20-day protocol), PHHs, HepaRG, HepG2, and human liver tissue was made using whole transcriptome RNA Seq analysis (Gupta et al. 2021). HLCs proved to be the closest cell system to PHHs, being the latter the closest to the in vivo situation. HepG2 was ranked as the least suitable model to predict and represent hepatic pathways, followed by HepaRG. However, in most studies to date, shorter protocols were applied to differentiate iPSCs into hepatocytes, resulting in immature models maintaining embryonic features (Choudhury et al. 2017; Li et al. 2019). Our study demonstrates that longer culture protocols (> 40 days) were required to obtain mature HLCs (Boon et al. 2020), with performing activities for various CYPs. At early stages of differentiation (20 days), HLCs still resemble an immature drug metabolism machinery, and therefore proved not to be relevant in drug metabolism studies. In sum, HLCs, regardless of their still lengthy culturing protocols, hold promise in studying drug metabolism, which can be applied in inter-individual response trials.

Our study provided a comprehensive characterization of the drug metabolism capacity across various in vitro hepatic models using multiple assays and approaches. Nevertheless, integration of different omics proved to be challenging. CYP gene expression was not always in accordance with CYP protein quantification. These findings may be explained by methodology constraints (e.g., differences in antibody specificity among CYP isoforms, used to perform the targeted proteomics), and/or differences in cellular copy number between transcript and protein (Wang et al. 2019). For example, *CYP1A2* transcript, detected in PHHs, failed to be quantified in any of the cell systems, nevertheless protein and activity were demonstrated for HepaRG, HepG2 and mHepG2. Furthermore, phase I metabolic activity was assessed by a well-established strategy of using drug metabolites as proxy for activity of selected CYP isoenzymes. A caveat with this approach is the known promiscuity—or sometimes classified as permissiveness—of CYP isoenzymes. It remains unclear whether the metabolic reaction happened via the challenged isoform, with protein levels below the detection threshold, or if another isoform facilitated it (Atkins 2020). Furthermore, CYP induction or inhibition, widely studied in the context of drug–drug interactions, is another confounding effect in studying isoform specificity (Gerets et al. 2012; Becker et al. 2021; Lee et al. 2024). Lastly, our LC–MS metabolomics approach, while proved to be robust and suitable for the detection of many putative metabolites, would require access to authentic standards and validation/quantification efforts to unambiguously assign and fully quantify drug metabolites. Regardless of these limitations, our multi-omics approach allowed for a comparative analysis of several cell systems and their potential use in drug metabolism studies, in a single study. Untargeted metabolomics opens a new door in the field, able to cover and follow a wider range of metabolic reactions toward a broader systems overview of drug metabolism.

In sum, our study highlights the strength of metabolic modulation in developing cellular metabolic machinery. Pertaining drug metabolism, both HLCs and mHepG2 are superior cell systems compared to traditional hepatocyte cell lines. Evidence was provided supporting that metabolic activity can be chemically boosted through nutrient regimens in in vitro systems, leading to better predictive cell models, with widespread application in metabolic disease and pharma development programs.

## Supplementary Information

Below is the link to the electronic supplementary material.

Supplementary file1 (PDF 3862 KB)

## Data Availability

Metabolomics, proteomics and qPCR data have been deposited in BioStudies (https://www.ebi.ac.uk/biostudies/) under the unique permanent identifiers S-RHER243, S-RHER246, S-RHER269, S-RHER270, S-RHER3 and are publicly available as of the date of publication. Raw data and/or any additional information will be shared by the corresponding author upon request.
