# Supplementary material for: Nutrient environment improves drug metabolic activity in human iPSC-derived hepatocytes and HepG2
Source: Arch Toxicol. 2025 Aug 12;99(11):4493–511. doi: 10.1007/s00204-025-04139-4 (PMC12477091; doi:10.1007/s00204-025-04139-4)
Supplement: Supplementary file 1 — Supplementary file1 (PDF 3862 KB) [file 204_2025_4139_MOESM1_ESM.pdf]

SUPPLEMENTARY INFORMATION

Nutrient environment improves drug metabolic activity in human iPSC-derived hepatocytes and HepG2

Victoria Pozo Garcia[1], Tuğçe S. Çobanoğlu[1], Helen Sophie Hammer[2], Rita Carlota[1], Kasper Holm[1], Catherine Verfaillie[3], Oliver Poetz[2,4], Paul Jennings[1], Sofia Moco[1]*

[1] Department of Chemistry and Pharmaceutical Sciences, Amsterdam Institute of Molecular and Life Sciences (AIMMS), Vrije Universiteit (VU) Amsterdam, De Boelelaan 1108, 1081 HZ Amsterdam, the Netherlands

[2] Signatope GmbH, Reutlingen, Germany

[3] Department of Development and Regeneration, Stem Cell Institute, KU Leuven, Leuven, Belgium

[4] NMI Natural and Medical Sciences Institute at the University of Tuebingen, Tuebingen, Germany

*Corresponding author email: s.moco@vu.nl

SUPPLEMENTARY FIGURES

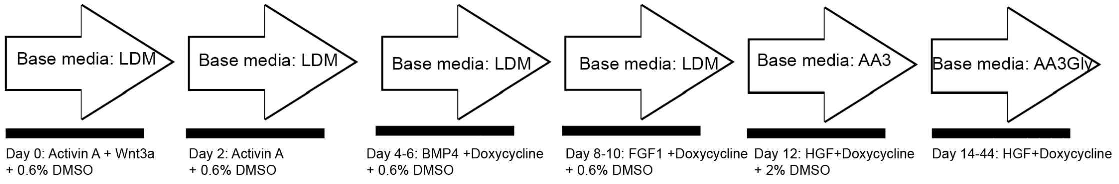

Fig. S1 Temporal scheme of HLCs differentiation process, indicating days of cytokine addition, and days of different media composition addition: LDM, AA3, and AA3Gly.

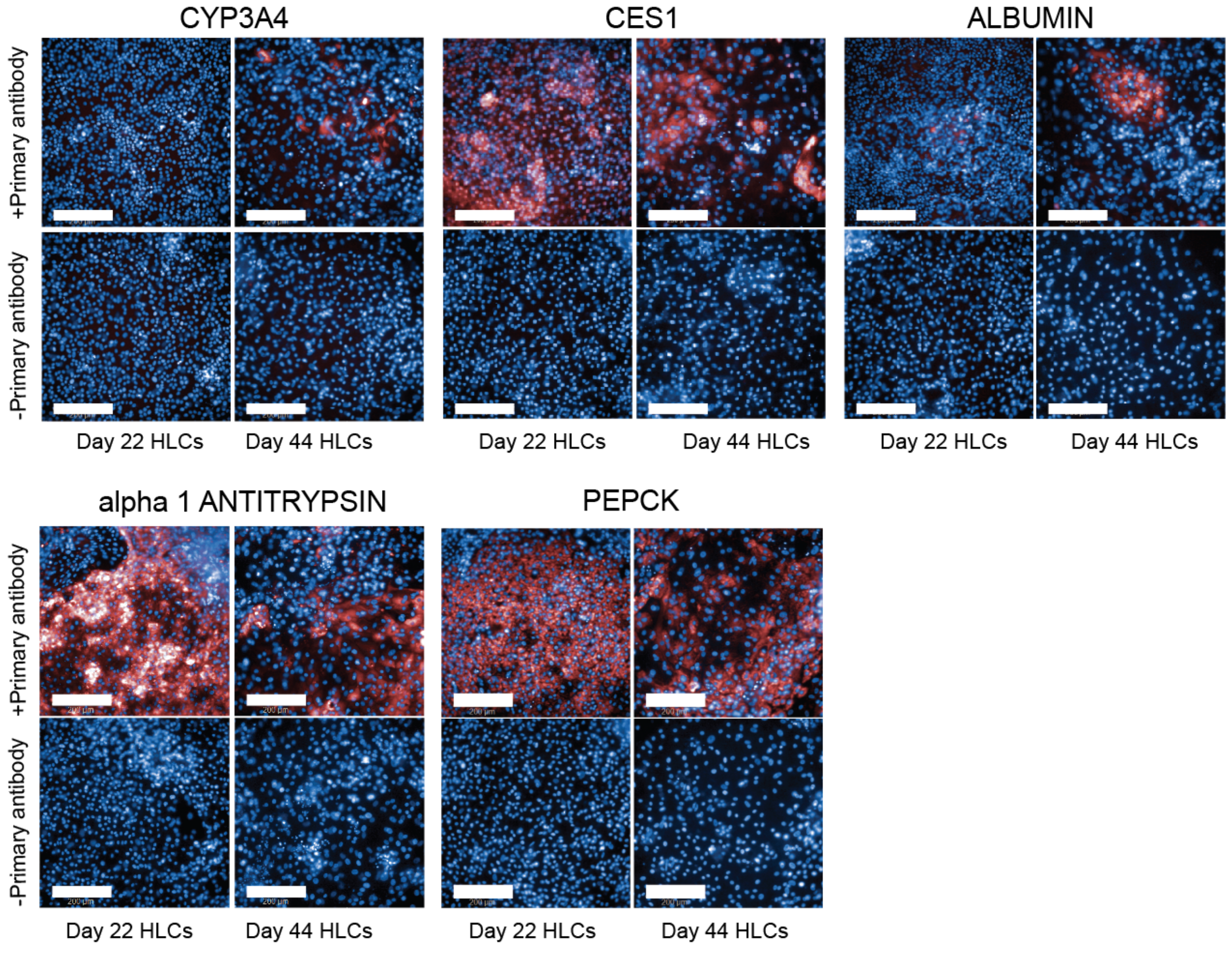

Fig. S2 CYP3A4, CES1, albumin, alpha 1 antitrypsin and PEPCK liver markers measured in day 22 HLCs and day 44 HLCs by immunofluorescence. Scale bar represents 200 µm. The cells were imaged using a 20x water immersion objective on the Operetta CLS High-Content Imager (PerkinElmer) with confocal imaging, and image analysis was performed with the Harmony 4.9 software. CES1, carboxylesterase 1; PEPCK, phosphoenolpyruvate carboxykinase.

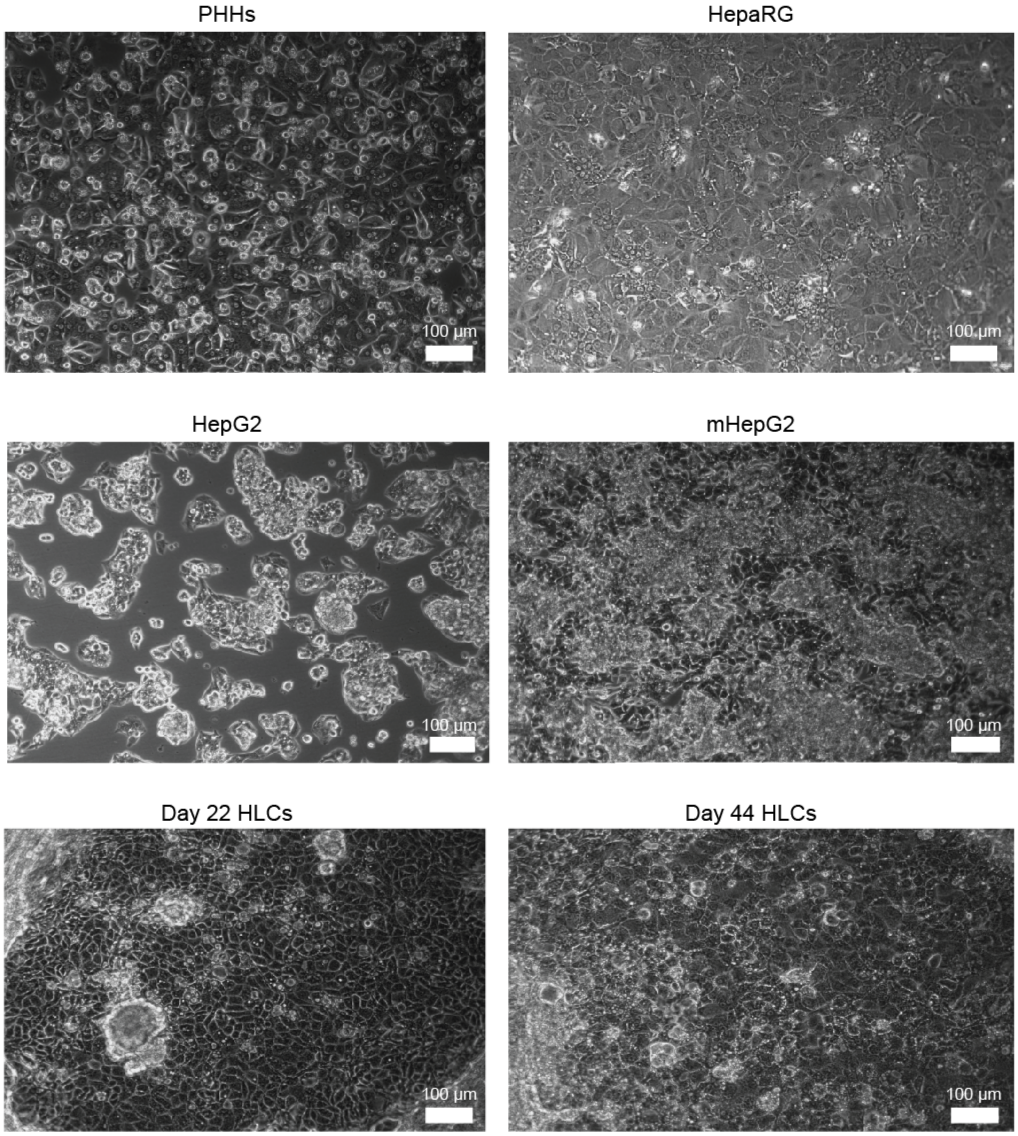

Fig. S3 Confocal pictures of all cell models used in the present study: PHHs, HepaRG, HepG2, mHepG2, day 22 HLCs, and day 44 HLCs. Objective used was 10x and the scale represents 100 µm.

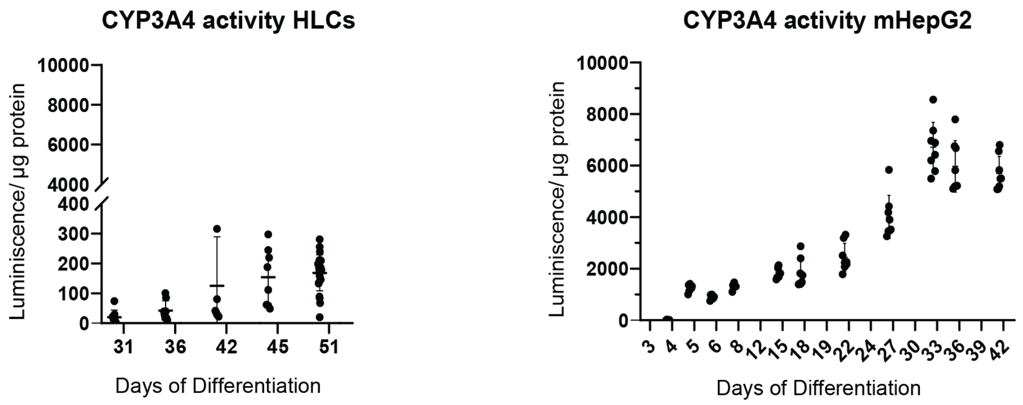

Fig. S4 CYP3A4 activity along HLCs and mHepG2 differentiation. Results are represented as Luminescence/µg protein, and the x-axis shows the days of differentiation of each cell model. N=8-24 replicates per cell model and time point.

SUPPLEMENTARY TABLES

Table S1 Materials used in cell culture.

Material	Supplier	Catalog number	CAS number
William's E Medium, GlutaMAX™ Supplement	Gibco, Landsmeer NL	32551020	
Hydrocortisone 21-hemisuccinate sodium salt	Merck Life Science, Amsterdam NL	H2270	125-04-2
Insulin solution human	Merck Life Science, Amsterdam NL	I9278	11061-68-0
Fetal Bovine Serum, qualified, Brazil	Gibco, Landsmeer NL	10270106	
DMEM, low glucose, pyruvate	Gibco, Landsmeer NL	31885023	
MEM amino acids solution (50X)	ThermoFisher, Landsmeer NL	15040033	
MEM non-essential amino acids solution (100X)	ThermoFisher, Landsmeer NL	12519059	
L-Glycine	Merck Life Science, Amsterdam NL	G7126	56-40-6
Trypsin-EDTA solution	Merck Life Science, Amsterdam NL	T4049	
Geltrex ™	Gibco, Landsmeer NL	A14133-02	
Gibco, Landsmeer NL™ Versene Solution/ EDTA	ThermoFisher, Landsmeer NL	15040033	
Penicillin-Streptomycin	Merck Life Science, Amsterdam NL	P4333	
DMSO for cell culture	Merck Life Science, Amsterdam NL	D8418	67-68-5
Gibco, Landsmeer NL™ StemPro™ Accutase™ Cell	ThermoFisher, Landsmeer NL	11599686	
Albumin, Bovine Serum, Fraction V, Fatty Acid-Free, Nuclease- and Protease-Free	Merck Life Science, Amsterdam NL	126609	

Table S2 Materials used for VU8 media preparation in iPSCs culture.

Material	Supplier	Catalog number	CAS number
DMEM, high glucose	Gibco, Landsmeer NL	41965-039	
Ham's F-12 Nutrient Mix	Gibco, Landsmeer NL	21765-029	
L-Ascorbic acid 2-phosphate	Merck Life Science, Amsterdam NL	A8960	1713265-25-8
Insulin	Gibco, Landsmeer NL	A11382II	

Transferrin	Merck Life Science, Amsterdam NL	T3705	11096-37-0
Sodium selenite	Merck Life Science, Amsterdam NL	S5261	10102-18-8
Recombinant FGF2-G3 protein	Qkine, Cambridge UK	Qk053	
Human Recombinant TGF-beta 1	StemCell Technologies, Saubt Egreve FR	78067.1	
GlutaMAX	Gibco, Landsmeer NL	35050-038	
Human Recombinant Albumin	Merck Life Science, Amsterdam NL	A9731	

Table S3 Materials used in LDM media preparation and cytokines used in HLCs differentiation.

Material	Supplier	Catalog number	CAS number
DMEM-Low glucose	Gibco, Landsmeer NL	31885	
MCDB 201 Medium	Merck Life Science, Amsterdam NL	M6770	
Penicillin-Streptomycin	Merck Life Science, Amsterdam NL	P4333	
L-Ascorbic acid 2-phosphate	Merck Life Science, Amsterdam NL	A8960	1713265-25-8
Insulin-Transferrin-Selenium (ITS -G) (100X)	Gibco, Landsmeer NL	4140-045	
Linoleic Acid-Albumin from bovine serum	Merck Life Science, Amsterdam NL	L9530	
2-Mercaptoethanol (50 mM)	Gibco, Landsmeer NL	31350010	
Dexamethasone-Water Soluble	Merck Life Science, Amsterdam NL	D-2915	50-02-2
Hydrocortisone 21-hemisuccinate sodium salt	Merck Life Science, Amsterdam NL	H2270	125-04-2
Recombinant mouse Wnt3a protein	Abcam, Amsterdam NL	ab81484	
Human Recombinant BMP-4	StemCell Technologies, Saubt Egreve FR	78211	
Human Recombinant HGF	StemCell Technologies, Saubt Egreve FR	78019.1	
Human/Mouse Recombinant Activin A	Stem cell technologies, Saubt Egreve FR	78001.1	
Human Recombinant FGF-acidic	StemCell Technologies, Saubt Egreve FR	78187.1	
Y-27632 dihydrochloride, Rho kinase inhibitor	Abcam, Amsterdam NL	Ab120129	
Doxycycline hydrochloride	Merck Life Science, Amsterdam NL	D3447	10592-13-9

Table S4 Materials used in primary human hepatocytes (PHHs) culture.

Material	Supplier	Catalog number
Biocoat Corning, Amsterdam NL Collagen I Cellware plates	Corning, Amsterdam NL	354408
LiverPool 10-donor Human hepatocytes 5M cells	BioIVT, Frankfurt am Main Germany	X008001
OptiThaw Hepatocyte Media	Tebu bio, Heerhugowaard NL	K8000
InVitroGRO CP medium	BioIVT, Frankfurt am Main Germany	Z99029
InVitroGRO HI medium	BioIVT, Frankfurt am Main Germany	Z99009

Table S5 Antibodies used for immunofluorescence.

Antibody	Supplier	Catalog number
CYP3A4	Abcam, Amsterdam NL	AB124921
Carboxylesterase 1 (CES1)	ThermoFisher, Landsmeer NL	PA5-19740
Albumin	Dako, Glostrup DK	A0001
Alpha 1 Antitrypsin	Dako, Glostrup DK	20079116
Phosphoenolpyruvate carboxykinase (PEPCK)	Merck Life Science, Amsterdam NL	SAB5701540
Hoechst 33342	ThermoFisher, Landsmeer NL	11534886
Alexa 647 donkey anti-rabbit IgG	ThermoFisher, Landsmeer NL	A32795

Table S6 Chemicals used to perform human liver microsomes (HLMs) incubations.

Chemical	Supplier	Catalog number	CAS number
D-Glucose 6-phosphate sodium salt	Merck Life Science, Amsterdam NL	G7879	54010-71-8
Glucose 6-phosphate dehydrogenase from *Leuconostoc mesenteroides*	Merck Life Science, Amsterdam NL	G8404	9001-40-5
NADP+	PROZOMIX, Haltwhistle UK	PRO-NADP	24292-60-2
Potassium phosphate monobasic	Merck Life Science, Amsterdam NL	P5655	7778-77-0
Potassium phosphate dibasic	Merck Life Science, Amsterdam NL	795496	7758-11-4
Magnesium chloride	Merck Life Science, Amsterdam NL	M8266	7786-30-3
EDTA	Merck Life Science, Amsterdam NL	EDS	60-00-4
Bupropion hydrochloride	TCI chemicals, Zwijndrecht NL	B3649	31677-93-7
Phenacetin	Merck Life Science, Amsterdam NL	77440	62-44-2
Rosiglitazone	TCI chemicals, Zwijndrecht NL	R0106	122320-73-4
Diclofenac sodium salt	Merck Life Science, Amsterdam NL	D6899	15307-79-6
Dextromethorphan	Merck Life Science, Amsterdam NL	D2531	6700-34-1
Chlorzoxazone	Merck Life Science, Amsterdam NL	C4397	95-25-0
Benzydamine hydrochloride	Merck Life Science, Amsterdam NL	B5524	132-69-4
Midazolam hydrochloride	Duchefa, Haarlem NL	-	59468-44-9
Coumarin	Merck Life Science, Amsterdam NL	C4261	91-64-5
7-Ethoxycoumarin	Merck Life Science, Amsterdam NL	E1379	31005-02-4
Meloxicam sodium salt	Merck Life Science, Amsterdam NL	M3935	71125-39-8

Table S7 Materials used for the qPCR reactions.

Material	Supplier	Catalog number
miRNeasy Mini Kit (50)	Qiagen, Venlo NL	217004
RNeasy Mini Kit (50)	Qiagen, Venlo NL	74104
iScript™ Reverse Transcription Supermix	Bio-Rad, Lunteren NL	1708841
Luna Universal qPCR Master Mix	BIOKE, Leiden NL	NEB M3003E

Table S8 Final amino acid concentration (mM) in HepG2, AAHepG2, LDM, AA3 and AA3Gly media.

Amino acid	HepG2	AAHepG2	LDM	AA3	AA3Gly
L-Glycine	0.4	268.0	0.2	1.5	267.9
L-Arginine hydrochloride	0.4	2.3	0.2	2.1	2.1
L-Cystine 2HCl	0.2	0.5	0.1	0.4	0.4
L-Glutamine	4.0	3.2	2.7	2.2	2.2
L-Histidine hydrochloride-H2O	0.2	0.8	0.1	0.7	0.7
L-Isoleucine	0.8	1.9	0.5	1.7	1.7
L-Leucine	0.8	1.9	0.5	1.7	1.7
L-Lysine hydrochloride	0.8	1.9	0.5	1.6	1.6
L-Methionine	0.2	0.5	0.1	0.4	0.4
L-Phenylalanine	0.4	1.0	0.2	0.8	0.8
L-Serine	0.4	1.6	0.2	1.5	1.5
L-Threonine	0.8	1.9	0.5	1.7	1.7
L-Tryptophan	0.1	0.2	0.0	0.2	0.2
L-Tyrosine disodium salt dihydrate	0.4	1.0	0.2	0.8	0.8
L-Valine	0.8	1.9	0.5	1.7	1.7
L-Alanine	0.0	1.3	0.0	1.3	1.3
L-Asparagine	0.0	1.3	0.0	1.3	1.3
L-Aspartic acid	0.0	1.3	0.0	1.3	1.3
L-Glutamic acid	0.0	1.3	0.0	1.3	1.3
L-Proline	0.0	1.3	0.0	1.3	1.3

Table S9 Composition of VU8 media.

Component	Final Concentration
DMEM, high glucose	/
Ham's F-12 Nutrient Mix	/
GlutaMAX	2 mM
L-Ascorbic acid 2-phosphate	200 µg/mL
ITS (Insulin-Transferrin-Sodium Selenite)	(15 µg/mL, 15 µg/mL, 15 ng/mL)
Human heat stable FGF2-G3	60 ng/mL
Human Recombinant TGF-beta 1	2 ng/mL
Glucose (already present in DMEM+F12)	17.5 mM
Sodium bicarbonate (already present in DMEM+F12)	29 M

Table S10 Composition of LDM media.

Component	Final Concentration
DMEM-Low glucose	57%
MCDB dissolved in 1 L H_2O pH-7.1	40%
Penicillin-Streptomycin	/
L-Ascorbic acid in H_2O	0.1 mM
Insulin-Transferrin-Selenium (ITS -G) (100X)	/
Linoleic Acid-Albumin from bovine serum albumin	/
2-Mercaptoethanol (50 mM)	0.05 mM
Dexamethasone in H_2O	1 µM
Hydrocortisone hemisuccinate in H_2O	10 µM

Table S11 Final concentration of cytokines in HLCs differentiation.

Cytokine	Final concentration in media
Recombinant mouse Wnt3a protein	0.05 µg/mL
Human Recombinant BMP-4	0.05 µg/mL
Human Recombinant HGF	0.02 µg/mL
Human/Mouse Recombinant Activin A	0.05 µg/mL
Human Recombinant FGF-acidic	0.02 µg/mL
Y-27632 dihydrochloride, Rho kinase inhibitor	10 µM
Doxycycline hydrochloride	5 µg/mL

Table S12 Primer sequences and threshold of fluorescence values (ΔR) set to quantify each gene.

Gene	Sequence 5'-3'	ΔR	Reference
CYP1A1	FW. GTCATCTGTGCCATTTGCTTTG	11	(Nishimura et al. 2002)
	RV. CAACCACCTCCCCGAAATTATT		
CYP1A2	FW. GACATCTTTGGAGCAGGATTTGA	22	(Choi et al. 2015)
	RV. CTTCCTCTGTATCTCAGGCTTGGT		
CYP2A6	FW. CCAGCACTTCCTGAATGAG	33	(Sun et al. 2022)
	RV. GGTGGTGAAGAAGAGAAAGAG		
CYP2B6	FW. AAACCAGACGCCTTCAATC	19	(Sun et al. 2022)
	RV. GACAAATCCGCTTCCCTAAG		
CYP2C8	FW. GGAAAACGAATTTGTGCAGGAG	38	(Chen et al. 2016)
	RV. GTGGCAGAGAAACAATCCCTT		
CYP2C9	FW. CGGATTTGTGTGGGAGAAG	25	(Sun et al. 2022)
	RV. CAACTGGAGTGGTGTCAAG		
CYP2C19	FW. CAACAACCCTCGGGACTTTA	12	(Sun et al. 2016)

	RV. GTCTCTGTCCCAGCTCCAAG		
CYP2D6	FW. TTCCTCAGGCTGCTGGAC	40	(Temesvári et al. 2012)
	RV. CGCTGGGATATGCAGGAG		
CYP2E1	FW. ATCGACCTCAGCCCTATAC	17	(Sun et al. 2022)
	RV. TGTCCTCCACACACTCAT		
CYP3A4	FW. GCCTGGTGCTCCTCTATCTA	28	(Sun et al. 2022)
	RV. GGCTGTTGACCATCATAAAAG		
CYP3A5	FW. ATG GAA AAA TGT GGG GAA CG	27	(Krusekopf et al. 2003)
	RV. CGC TGG TGA AGG TTG GAG AC		
Alpha-Tubulin	FW. CTC CTT GCC AAT GGT GTA GTG C	13	OriGene
	RV. CGG GCA GTG TTT GTA GAC TTG G		
GAPDH	FW. GTC TCC TCT GAC TTC AAC AGC G	14	OriGene
	RV. ACC ACC CTG TTG CTG TTG CTG TAG CCA A		

Table S13 List of metabolites found in literature from the tested CYP probes (in bold).

Monoisotopic mass (MIM); Molecular formula (MF)

**Metabolite name in literature**	**MF**	**MIM**	**Reference**
**7-Ethoxycoumarin**	C11H10O3	190.0629942	(Feng et al. 2018)
7-Ethoxy-4-hydroxycoumarin	C11H10O4	206.0579088	
7-Ethoxy-3-hydroxycoumarin	C11H10O4	206.0579088	
7-Hydroxycoumarin	C9H6O3	162.0316941	
Coumarin	C9H6O2	146.0367794	
Coumarin-7-*O*-sulfate	C9H6O6S	241.9885091	
Coumarin-7-*O*-glucuronide	C15H14O9	338.063782	
7-Ethoxycoumarin-3-glucuronide	C17H18O10	382.089968	
7,X-Dihydroxycoumarin	C9H6O4	178.0266087	
7,X-Dihydroxycoumarin glucuronide	C15H14O10	354.0586966	
M15	C10H8O4	192.0422587	
M12	C9H6O7S	257.9834237	
M2	C15H16O8	324.0845175	
M16	C11H12O4	208.0735589	
M5	C11H12O5	224.0684735	
7-Ethoxycoumarin 3,4-epoxide	C11H10O4	206.0579088	
M20	C21H25N3O9S	495.1311506	
M17, M26	C14H15NO5S	309.0670938	
M22	C10H12O4	196.0735589	
M25	C10H12O7S	276.0303739	
M19	C10H12O3	180.0786442	
M23	C10H14O3	182.0942943	
M10	C16H20O10	372.1056468	
M9	C16H20O10	372.1056468	
M11	C16H22O9	358.1263823	
M8	C16H22O9	358.1263823	
**Benzydamine**	C19H23N3O	309.1841124	(Santi et al. 2002; Fisher et al. 2002; Taniguchi-Takizawa et al. 2015)
Benzydamine *N*-oxide	C19H23N3O2	325.179027	
Norbenzydamine	C18H21N3O	295.1684623	
Benzydamine carboxylic acid	C17H16N2O3	296.1160924	
1-Benzyl-1H-indazol-3-ol	C14H12N2O	224.094963	
3-((1H-Indazol-3-yl)oxy)-*N,N*-demethylpropan-1-amine	C12H17N3O	219.1371622	
Benzydamine hydroxide	C19H23N3O2	325.179027	
Benzydamine glucuronide	C25H31N3O8	501.211115	
Benzydamine *N*-glucuronide	C25H32N3O8+	502.21894	

Benzydamine *N*-oxide hydroxide	C19H23N3O3	341.1739416	
Benzydamine *N*-oxide *N*-glucuronide	C25H32N3O9+	518.2138546	
Norbenzydamine *N*-glucuronide	C24H30N3O7+	472.2083753	
3-((1H-Indazol-3-yl)oxy)-*N,N*-demethylpropan-1-amine glucuronide	C18H25N3O7	395.1692501	
1-Benzyl-1H-indazol-3-ol glucuronide	C20H20N2O7	400.127051	
Norbenzydamine glucuronide	C24H29N3O7	471.2005503	
Norbenzydamine hydroxide	C18H21N3O2	311.1633769	
**Bupropion**	C13H18ClNO	239.1076919	(Chen et al. 2010; Gufford et al. 2016; Costa et al. 2019)
Hydroxybupropion	C13H18ClNO2	255.1026065	
4-Hydroxybupropion	C13H18ClNO2	255.1026065	
3-Hydroxybupropion	C13H18ClNO2	255.1026065	
Threohydrobupropion	C13H20ClNO	241.1233419	
4-Hydroxybupropion glucuronide	C19H26ClNO8	431.1346945	
4-Hydroxybupropion sulfate	C13H18ClNO5S	335.0594215	
Threohydrobupropion glucuronide	C19H28ClNO7	417.1554299	
Erythrohydrobupropion glucuronide	C19H28ClNO7	417.1554299	
Threo-4-hydroxy-hydrobupropion	C13H20ClNO2	257.1182566	
Hydroxybupropion threoketal	C13H18ClNO2	255.1026065	
meta-Chlorobenzoic acid	C7H5ClO2	155.9978071	
meta-Chlorohippuric acid	C11H12ClNO3	241.0505709	
**Chlorzoxazone**	C7H4ClNO2	168.993056	(Quesnot et al. 2018)
6-Hydroxychlorzoxazone	C7H4ClNO3	184.9879707	
Chlorzoxazone *O*-glucuronide	C13H12ClNO9	361.0200586	
Chlorzoxazone *N*-glucuronide	C13H12ClNO8	345.025144	
Hydroxychlorzoxazone *N*-glucuronide	C13H12ClNO9	361.0200586	
**Coumarin**	C9H6O2	146.0367794	(Lake et al. 1992; Lake 1999; Vassallo et al. 2003; Feng et al. 2018; Hsieh et al. 2019; Pitaro et al. 2022)
7-Hydroxycoumarin	C9H6O3	162.0316941	
3-Hydroxycoumarin	C9H6O3	162.0316941	
Coumarin-3,4-epoxide	C9H6O3	162.0316941	
6,7-Dihydroxycoumarin	C9H6O4	178.0266087	
3,4-Dihydrocoumarin	C9H8O2	148.0524295	
ortho-Hydroxyphenylpropionic acid	C9H10O3	166.0629942	
ortho-Coumaric acid	C9H8O3	164.0473441	
4-Hydroxycoumarin	C9H6O3	162.0316941	
5-, 6- and 8-Hydroxy coumarin	C9H6O3	162.0316941	
Coumarin glucuronide	C15H14O9	338.063782	
Coumarin sulphate	C9H6O6S	241.9885091	
ortho-Hydroxyphenyl-acetaldehyde (ortho-HPA)	C8H8O2	136.0524295	
Coumarin glutathione conjugate (1)	C19H21N3O8S	451.1049358	
Coumarin glutathione conjugate (2)	C19H23N3O9S	469.1155005	
Coumarin 3-mercapturic acid (1)	C14H15NO6S	325.0620084	
Coumarin 3-mercapturic acid (2)	C14H13NO5S	307.0514437	
ortho-Hydroxyphenylethanol (ortho-HPE)	C8H10O2	138.0680796	
ortho-Hydroxyphenylacetic acid (ortho-HPAA)	C8H8O3	152.0473441	
ortho-Hydroxyphenyllactic acid (ortho-HPLA)	C9H10O4	182.0579088	
**Dextromethorphan**	C18H25NO	271.1936144	(Lutz et al. 2004; Taylor et al. 2016)
Dextrorphan	C17H23NO	257.1779644	
Dextrorphan-*O*-glucuronide	C23H31NO7	433.2100523	
Dextrorphan sulfate	C17H23NO4S	337.1347794	
3-Methoxymorphinan	C17H23NO	257.1779644	
3-Hydroxymorphinan	C16H21NO	243.1623143	
3-Hydroxymorphinan sulfate	C16H21NO3S	307.1242147	
3-Hydroxymorphinan glucuronide	C22H29NO7	419.1944023	
3-Methoxymorphinan *N*-glucuronide	C23H31NO7	433.2100523	
**Diclofenac**	C14H11Cl2NO2	295.0166839	(Poon et al. 2001; Boelsterli 2003;
4-Hydroxydiclofenac	C14H11Cl2NO3	311.0115986	

3-Hydroxydiclofenac	C14H11Cl2NO3	311.0115986	Boerma et al. 2014; Kamimura et al. 2015)
5-Hydroxydiclofenac	C14H11Cl2NO3	311.0115986	
4,5-Dihydroxydiclofenac	C14H11Cl2NO4	327.0065132	
Diclofenac-2,5-quinone imine	C14H9Cl2NO3	308.9959485	
Diclofenac acyl glucuronide	C20H19Cl2NO8	471.0487719	
3,4-Dihydroxydiclofenac	C14H11Cl2NO4	327.0065132	
5,*N*-Dihydroxydiclofenac	C14H13Cl2NO4	329.0221632	
Diclofenac *O*-imine methine	C13H11Cl2N	251.0268547	
Diclofenac 2,3-oxide	C14H11Cl2NO3	311.0115986	
Diclofenac radical	C14H11Cl2NO2+	295.0166839	
4-Hydroxydiclofenac quinone imine	C14H11Cl2NO3	311.0115986	
4-Hydroxy-2-glutathione conjugated monoclofenac	C24H27ClN4O9S	582.1187273	
4-Hydroxy-3-glutathione conjugated monoclofenac	C24H26Cl2N4O9S	616.0797549	
5-Hydroxy-6-glutathione conjugated diclofenac	C24H26Cl2N4O9S	616.0797549	
5-Hydroxy-4-glutathione conjugated diclofenac	C24H26Cl2N4O9S	616.0797549	
2-(2,6-Dichlorophenylamino)-benzyl-*S*-thioether	C23H26Cl2N4O6S	556.0950111	
3-Hydroxy-2-glutathione conjugated monoclofenac	C24H29ClN4O9S	584.1343773	
2-Hydroxy-3-glutathione conjugated diclofenac	C24H28Cl2N4O9S	618.095405	
2-Hydroxy-3-glutathione conjugated diclofenac	C24H29ClN4O9S	584.1343773	
2-(Glutathione-*S*-yl)-deschloro-diclofenac	C24H29ClN4O8S	568.1394627	
3-Hydroxy-4-methoxydiclofenac	C15H13Cl2NO4	341.0221632	
5-Hydroxy-4-mercapturic acid conjugated diclofenac	C19H18Cl2N2O6S	472.0262628	
4-Hydroxy-3-mercapturic acid conjugated monoclofenac	C19H18Cl2N2O6S	472.0262628	
5-Hydroxy-6-mercapturic acid conjugated diclofenac	C19H18Cl2N2O6S	472.0262628	
2-(Mercapturic acid)-deschloro-diclofenac	C19H19ClN2O5S	422.0703205	
4-Hydroxy-2-mercapturic acid conjugated monoclofenac	C19H19ClN2O6S	438.0652352	
2-Hydroxy-3-mercapturic acid conjugated diclofenac	C19H21ClN2O6S	440.0808852	
3-Hydroxy-2-mercapturic acid conjugated diclofenac	C19H21ClN2O6S	440.0808852	
2-(2,6-Dichlorophenylamino)-benzyl-*S*-thioether	C18H18Cl2N2O3S	412.0415189	
**Midazolam**	C18H13ClFN3	325.0782032	(Zhu et al. 2008; Seo et al. 2010; Nguyen et al. 2016)
1-Hydroxymidazolam	C18H13ClFN3O	341.0731179	
4-Hydroxymidazolam	C18H13ClFN3O	341.0731179	
N-Midazolam glucuronide	C24H22ClFN3O6+	502.1181163	
Midazolam 4-glucuronide	C24H21ClFN3O7	517.1052058	
Midazolam 1-glucuronide	C24H21ClFN3O7	517.1052058	
1,4-Dihydroxymidazolam	C18H13ClFN3O2	357.0680325	
1-Hydroxymidazolam *N*-glucuronide	C24H22ClFN3O7+	518.1130309	
4-Hydroxymidazolam *N*-glucuronide	C24H22ClFN3O7+	518.1130309	
1,4-Dihydroxymidazolam *N*-glucuronide	C24H22ClFN3O9+	550.1028601	
1,4-Dihydroxymidazolam 4-glucuronide	C24H21ClFN3O8	533.1001205	
1,4-Dihydroxymidazolam 1-glucuronide	C24H21ClFN3O8	533.1001205	
**Phenacetin**	C10H13NO2	179.0946287	(McLean 1978; Hinson 1983; Kudo et al. 2000; Hodgman and Garrard 2012)
Acetaminophen	C8H9NO2	151.0633285	
Phenetidine	C8H11NO	137.084064	
N-(4-(2-Hydroxyethoxy) phenyl) acetamide (Hydroxy phenacetin)	C10H13NO3	195.0895433	
4-Acetaminophenoxyacetic acid	C10H11NO4	209.0688078	
Acetaminophen glucuronide	C14H17NO8	327.0954165	
Acetaminophen sulfate	C8H9NO5S	231.0201436	
N-Acetyl-para-benzoquinone imine	C8H7NO2	149.0476785	
Acetaminophen catechol	C8H9NO3	167.0582431	
Quinone	C6H4O2	108.0211294	
Quinol	C6H6O2	110.0367794	
Acetamide	C2H5NO	59.03711378	
Phenol sulfate	C6H6O5S	189.9935945	
Phenol glucuronide	C12H14O8	286.0688674	
Acetaminophen glutathione conjugate	C18H24N4O8S	456.1314849	

Acetaminophen mercapturic conjugate	C13H16N2O5S	312.0779928	
**Rosiglitazone**	C18H19N3O3S	357.1147126	(Cox et al. 2000; Hruska et al. 2005)
N-Desmethylrosiglitazone	C17H17N3O3S	343.0990626	
N-Despyridinyl rosiglitazone	C13H16N2O3S	280.0881635	
Para-Hydroxy rosiglitazone	C18H19N3O4S	373.1096273	
Ortho-Hydroxy rosiglitazone	C18H19N3O4S	373.1096273	
N-Desmethyl-para-hydroxy rosiglitazone	C17H17N3O4S	359.0939772	
N-Desmethyl-ortho-hydroxy rosiglitazone	C17H17N3O4S	359.0939772	
Phenoxyacetic acid derivative of rosiglitazone	C12H11NO5S	281.0357936	
N-Desmethyl glucuronide rosiglitazone	C23H25N3O9S	519.1311506	
N-Desmethyl-para-*O*-sulfate rosiglitazone	C17H17N3O7S2	439.0507922	
Para-*O*-sulfate rosiglitazone	C18H19N3O7S2	453.0664423	
Para-*O*-glucuronide rosiglitazone	C24H27N3O10S	549.1417152	
N-Desmethyl-ortho-*O*-sulfate rosiglitazone	C17H17N3O7S2	439.0507922	
Ortho-*O*-sulfate rosiglitazone	C18H19N3O7S2	453.0664423	
Ortho-*O*-glucuronide rosiglitazone	C24H27N3O10S	549.1417152	
N-Desmethylrosiglitazone ortho-*O*-glucuronide	C23H25N3O10S	535.1260652	
N-Desmethylrosiglitazone para-*O*-glucuronide	C23H25N3O10S	535.1260652	
Rosiglitazone glucuronide	C24H27N3O9S	533.1468006	

Table S14 Putative identified metabolites across all tested *in vitro* models (HLMs, HepG2, HepaRG, mHepG2 and HLCs) when incubated with model drugs (7-ethoxycoumarin, benzydamine, coumarin, diclofenac, rosiglitazone, bupropion, midazolam, dextromethorphan, phenacetin and chlorzoxazone) analyzed using LC-MS.

In this table it is reported the experimental m/z value, mass error in ppm, retention time (in minutes), ion polarity in which metabolites were detected, and molecular formula. All identified metabolites were present in drug-incubated samples, absent in control and absent or higher than in blank samples.

**Metabolite common name**	**Polarity**	**Rt**	**Found**	**ppm**	**MF**	**HLMs**	**HepG2**	**HepaRG**	**mHepG2**	**HLCs**	**Standard**
**7-Ethoxycoumarin**	positive	13.6	191.0698	2.4	C11H10O3	x	x	x	x	x	x
7-Hydroxycoumarin	positive	7.8	163.0389	0.4	C9H6O3	x	x		x	x	x
Coumarin sulfate	negative	4.3	240.9819	2.7	C9H6O6S		x	x	x	x	
Dihydroxycoumarin 1	negative	4.4	177.0192	0.7	C9H6O4	x					
Dihydroxycoumarin 2	negative	6.0	177.0192	0.7	C9H6O4	x					
Coumarin metabolite 1	negative	9.7	195.0668	2.6	C10H12O4	x		x	x		
Coumarin metabolite 2	negative	9.8	181.0864	3.4	C10H14O3	x					
**Benzydamine**	positive	12.3	310.1907	2.2	C19H23N3O	x	x	x	x	x	x
3-((1H-Indazol-3-yl)oxy)-*N,N*-demethylpropan-1-amine	positive	6.7	220.1442	1.0	C12H17N3O	x	x	x	x	x	
Benzydamine glucuronide	positive	6.7	502.2172	2.3	C25H31N3O8				x		
Norbenzydamine	positive	12.0	296.1743	4.8	C18H21N3O	x	x	x	x	x	
Benzydamine oxide	positive	12.3	326.1859	1.2	C19H23N3O2	x	x	x	x	x	
Benzydamine oxide hydroxide	positive	10.5	342.1817	1.4	C19H23N3O3	x			x		
Norbenzydamine hydroxide 1	positive	10.0	312.1693	4.3	C18H21N3O2	x			x		
Norbenzydamine hydroxide 2	positive	9.8	312.1703	1.1	C18H21N3O2				x		
Benzydamine hydroxide	positive	10.1	326.1856	2.1	C19H23N3O2	x	x		x	x	
1-Benzyl-1H-indazol-3-ol	positive	13.2	225.1025	1.1	C14H12N2O	x			x		
**Coumarin**	positive	10.1	147.044	0.3	C9H6O2	x	x	x	x	x	x
Coumarin epoxide	positive	8.3	163.0389	0.4	C9H6O3	x					
Hydroxycoumarin	negative	6.6	161.0239	3.2	C9H6O3	x					
Coumarin sulfate	negative	4.3	240.9819	2.7	C9H6O6S				x	x	
7-Hydroxycoumarin	negative	7.8	161.0247	1.7	C9H6O3	x			x	x	x
Coumaric acid	negative	9.3	163.0400	0.4	C9H8O3					x	
Dihydroxycoumarin 1	negative	4.4	177.0192	0.7	C9H6O4	x					
Dihydroxycoumarin 2	negative	6.0	177.0192	0.7	C9H6O4	x					
Hydroxyphenylacetic acid	negative	6.9	151.0396	3.1	C8H8O3	x	x	x	x		
Hydroxyphenylethanol	negative	7.0	137.0605	2.2	C8H10O2	x					

**Diclofenac**	positive	17.2	296.024	0.1	C14H11Cl2NO2	x	x	x	x	x	x
Hydroxydiclofenac	positive	14.3	312.0183	1.8	C14H11Cl2NO3	x		x	x	x	
Diclofenac quinone imine	negative	14.6	310.0029	1.0	C14H11Cl2NO3	x		x	x		
**Rosiglitazone**	positive	9.0	358.1208	3.3	C18H19N3O3S	x	x	x	x	x	x
Hydroxyrosiglitazone	positive	8.6	374.1156	3.4	C18H19N3O4S	x		x	x	x	
Desmethylrosiglitazone	positive	8.1	344.1049	4.1	C17H17N3O3S	x	x	x	x	x	
Rosiglitazone-*O*-sulfate	negative	8.8	452.0607	3.3	C18H19N3O7S2				x	x	
Despyridinyl rosiglitazone	positive	3.8	281.0967	4.4	C13H16N2O3S	x	x	x	x	x	
Desmethyl hydroxy rosiglitazone 1	positive	7.9	360.1018	1.5	C17H17N3O4S	x					
Desmethyl hydroxy rosiglitazone 2	positive	8.1	360.0995	4.8	C17H17N3O4S	x					
**Bupropion**	positive	9.6	240.1138	4.8	C13H18ClNO	x	x	x	x	x	x
Hydroxybupropion 1	positive	8.2	256.1093	2.2	C13H18ClNO2	x		x	x	x	
Hydroxybupropion 2	negative	7.7	254.095	1.3	C13H18ClNO2	x	x	x		x	
Hydroxybupropion 3	negative	8.9	254.0948	2.0	C13H18ClNO2	x	x	x		x	
Threohydrobupropion 1	positive	9.8	242.1305	0.4	C13H20ClNO	x	x	x	x	x	
Threohydrobupropion 2	positive	9.5	242.131	1.5	C13H20ClNO	x	x	x	x	x	
Chlorobenzoic acid	negative	12.3	154.9902	2.1	C7H5ClO2				x	x	
Threo- hydroxy-hydrobupropion 1	positive	6.8	258.1256	0.2	C13H20ClNO2	x					
Threo- hydroxy-hydrobupropion 2	positive	8.6	258.1268	4.9	C13H20ClNO2	x					
Threo-hydroxy-hydrobupropion 3	positive	8.8	258.1261	2.1	C13H20ClNO2	x					
**Midazolam**	positive	11.0	326.0852	0.8	C18H13ClFN3	x	x	x	x	x	x
Hydroxymidazolam 1	positive	10.2	342.0793	3.2	C18H13ClFN3O	x	x		x	x	
Hydroxymidazolam 2	positive	10.7	342.079	4.0	C18H13ClFN3O	x	x	x	x	x	
Dihydroxymidazolam	positive	9.9	358.0769	4.4	C18H13ClFN3O2	x					
**Dextromethorphan**	positive	10.9	272.2015	2.2	C18H25NO	x	x	x	x	x	x
Dextrorphan	positive	7.7	258.185	0.9	C17H23NO	x	x	x	x	x	
Hydroxymorphinan	positive	7.6	244.1694	0.7	C16H21NO	x			x		
Methoxymorphinan	positive	10.8	258.1857	1.7	C17H23NO	x		x			
**Phenacetin**	positive	9.9	180.1026	3.8	C10H13NO2	x	x	x	x	x	x
Acetaminophen	positive	2.1	152.0705	0.6	C8H9NO2	x	x	x	x		x
Hydroxyphenacetin	positive	8.9	196.0977	4.4	C10H13NO3	x					
**Chlorzoxazone**	negative	11.4	167.9858	0.1	C7H4ClNO2	x	x	x	x	x	x
Chlorzoxazone glucuronide	negative	7.4	344.0194	4.4	C13H12ClNO8				x		
Hydroxychlorzoxazone	negative	6.1	183.9813	3.2	C7H4ClNO3	x			x		

References

Boelsterli UA (2003) Diclofenac-induced liver injury: a paradigm of idiosyncratic drug toxicity. Toxicol Appl Pharmacol 192:307–22. https://doi.org/10.1016/s0041-008x(03)00368-5

Boerma JS, Vermeulen NPE, Commandeur JNM (2014) One-electron oxidation of diclofenac by human cytochrome P450s as a potential bioactivation mechanism for formation of 2’-(glutathion-S-yl)-deschloro-diclofenac. Chem Biol Interact 207:32–40. https://doi.org/10.1016/j.cbi.2013.11.001

Chen Y, Huang W, Chen F, et al (2016) Pregnane X receptors regulate CYP2C8 and P-glycoprotein to impact on the resistance of NSCLC cells to Taxol. Cancer Med 5:3564–3571. https://doi.org/10.1002/cam4.960

Chen Y, Liu H, Liu L, et al (2010) The in vitro metabolism of bupropion revisited: concentration dependent involvement of cytochrome P450 2C19. Xenobiotica 40:536–46. https://doi.org/10.3109/00498254.2010.492880

Choi JM, Oh SJ, Lee SY, et al (2015) HepG2 cells as an in vitro model for evaluation of cytochrome P450 induction by xenobiotics. Arch Pharm Res 38:691–704. https://doi.org/10.1007/s12272-014-0502-6

Costa R, Oliveira NG, Dinis-Oliveira RJ (2019) Pharmacokinetic and pharmacodynamic of bupropion: integrative overview of relevant clinical and forensic aspects. Drug Metab Rev 51:293–313. https://doi.org/10.1080/03602532.2019.1620763

Cox PJ, Ryan DA, Hollis FJ, et al (2000) Absorption, disposition, and metabolism of rosiglitazone, a potent thiazolidinedione insulin sensitizer, in humans. Drug Metab Dispos 28:772–80

Feng W-Y, Wen J, Stauber K (2018) In vitro Drug Metabolism Investigation of 7-Ethoxycoumarin in Human, Monkey, Dog and Rat Hepatocytes by High Resolution LC-MS/MS. Drug Metab Lett 12:33–53. https://doi.org/10.2174/1872312812666180418142056

Fisher MB, Yoon K, Vaughn ML, et al (2002) Flavin-Containing Monooxygenase Activity in Hepatocytes and Microsomes: In Vitro Characterization and In Vivo Scaling of Benzydamine Clearance. Drug Metabolism and Disposition 30:1087–1093. https://doi.org/10.1124/dmd.30.10.1087

Gufford BT, Lu JBL, Metzger IF, et al (2016) Stereoselective glucuronidation of bupropion metabolites in vitro and in vivo. Drug Metabolism and Disposition 44:544–553. https://doi.org/10.1124/dmd.115.068908

Hinson JA (1983) Reactive metabolites of phenacetin and acetaminophen: a review. Environ Health Perspect 49:71–9. https://doi.org/10.1289/ehp.834971

Hodgman MJ, Garrard AR (2012) A Review of Acetaminophen Poisoning. Crit Care Clin 28:499–516

Hruska MW, Amico JA, Langaee TY, et al (2005) The effect of trimethoprim on CYP2C8 mediated rosiglitazone metabolism in human liver microsomes and healthy subjects. Br J Clin Pharmacol 59:70–79. https://doi.org/10.1111/j.1365-2125.2005.02263.x

Hsieh CYJ, Sun M, Osborne G, et al (2019) Cancer Hazard Identification Integrating Human Variability: The Case of Coumarin. Int J Toxicol 38:501–552

Kamimura H, Ito S, Nozawa K, et al (2015) Formation of the accumulative human metabolite and human-specific glutathione conjugate of diclofenac in TK-NOG chimeric mice with humanized livers. Drug Metabolism and Disposition 43:309–316. https://doi.org/10.1124/dmd.114.061689

Krusekopf S, Roots I, Kleeberg U (2003) Differential drug-induced mRNA expression of human CYP3A4 compared to CYP3A5, CYP3A7 and CYP3A43. Eur J Pharmacol 466:7–12. https://doi.org/10.1016/S0014-2999(03)01481-X

Kudo S, Umehara K, Hosokawa M, et al (2000) Phenacetin deacetylase activity in human liver microsomes: distribution, kinetics, and chemical inhibition and stimulation. J Pharmacol Exp Ther 294:80–8

Lake BG (1999) Coumarin metabolism, toxicity and carcinogenicity: relevance for human risk assessment. Food Chem Toxicol 37:423–53. https://doi.org/10.1016/s0278-6915(99)00010-1

Lake BG, Osborne DJ, Walters DG, Price RJ (1992) Identification of o-hydroxyphenylacetaldehyde as a major metabolite of coumarin in rat hepatic microsomes. Food and Chemical Toxicology 30:99–104. https://doi.org/10.1016/0278-6915(92)90144-A

Lutz U, Völkel W, Lutz RW, Lutz WK (2004) LC-MS/MS analysis of dextromethorphan metabolism in human saliva and urine to determine CYP2D6 phenotype and individual variability in N-demethylation and glucuronidation. J Chromatogr B Analyt Technol Biomed Life Sci 813:217–225. https://doi.org/10.1016/j.jchromb.2004.09.040

McLean S (1978) Metabolism of phenacetin and N-hydroxyphenacetin in isolated rat hepatocytes. Naunyn Schmiedebergs Arch Pharmacol 305:173–80. https://doi.org/10.1007/BF00508289

Nguyen HQ, Kimoto E, Callegari E, Obach RS (2016) Mechanistic Modeling to Predict Midazolam Metabolite Exposure from In Vitro Data. Drug Metab Dispos 44:781–91. https://doi.org/10.1124/dmd.115.068601

Nishimura M, Yoshitsugu H, Naito S, Hiraoka I (2002) Evaluation of gene induction of drug-metabolizing enzymes and transporters in primary culture of human hepatocytes using high-sensitivity real-time reverse transcription PCR. Yakugaku Zasshi 122:339–61. https://doi.org/10.1248/yakushi.122.339

Pitaro M, Croce N, Gallo V, et al (2022) Coumarin-Induced Hepatotoxicity: A Narrative Review. Molecules 27:. https://doi.org/10.3390/molecules27249063

Poon GK, Chen Q, Teffera Y, et al (2001) Bioactivation of diclofenac via benzoquinone imine intermediates-identification of urinary mercapturic acid derivatives in rats and humans. Drug Metab Dispos 29:1608–13

Quesnot N, Bucher S, Gade C, et al (2018) Production of chlorzoxazone glucuronides via cytochrome P4502E1 dependent and independent pathways in human hepatocytes. Arch Toxicol 92:3077–3091. https://doi.org/10.1007/s00204-018-2300-2

Santi A, Anfossi P, Coldham NG, et al (2002) Biotransformation of benzydamine by microsomes and precision-cut slices prepared from cattle liver. Xenobiotica 32:73–86. https://doi.org/10.1080/00498250110085827

Seo KA, Bae SK, Choi YK, et al (2010) Metabolism of 1′- and 4-hydroxymidazolam by glucuronide conjugation is largely mediated by UDP-glucuronosyltransferases 1A4, 2B4, and 2B7. Drug Metabolism and Disposition 38:2007–2013. https://doi.org/10.1124/dmd.110.035295

Sun H, Lou XY, Wu XY, et al (2016) Up-regulation of CYP2C19 expression by BuChang NaoXinTong via PXR activation in HepG2 cells. PLoS One 11:. https://doi.org/10.1371/journal.pone.0160285

Sun X, He L, Bi H, et al (2022) Prenatal ethanol exposure induces dynamic changes of expression and activity of hepatic cytochrome P450 isoforms in male rat offspring. Reproductive Toxicology 109:101–108. https://doi.org/10.1016/j.reprotox.2022.03.002

Taniguchi-Takizawa T, Shimizu M, Kume T, Yamazaki H (2015) Benzydamine N-oxygenation as an index for flavin-containing monooxygenase activity and benzydamine N-demethylation by cytochrome P450 enzymes in liver microsomes from rats, dogs, monkeys, and humans. Drug Metab Pharmacokinet 30:64–69. https://doi.org/10.1016/j.dmpk.2014.09.006

Taylor CP, Traynelis SF, Siffert J, et al (2016) Pharmacology of dextromethorphan: Relevance to dextromethorphan/quinidine (Nuedexta®) clinical use. Pharmacol Ther 164:170–182. https://doi.org/10.1016/j.pharmthera.2016.04.010

Temesvári M, Kóbori L, Paulik J, et al (2012) Estimation of drug-metabolizing capacity by cytochrome P450 genotyping and expression. Journal of Pharmacology and Experimental Therapeutics 341:294–305. https://doi.org/10.1124/jpet.111.189597

Vassallo JD, Morrall SW, Fliter KL, et al (2003) Liquid chromatographic determination of the glutathione conjugate and ring-opened metabolites formed from coumarin epoxidation. J Chromatogr B Analyt Technol Biomed Life Sci 794:257–271. https://doi.org/10.1016/S1570-0232(03)00473-2

Zhu B, Bush D, Doss GA, et al (2008) Characterization of 1′-hydroxymidazolam glucuronidation in human liver microsomes. Drug Metabolism and Disposition 36:331–338. https://doi.org/10.1124/dmd.107.017962

OriGene. Lentiviral products - Lenti shRNA & Lenti-ORF. OriGene Technologies, Inc.
